# A Treatise on the Diagnosis and Treatment of Diseases of the Chest. Part I. Diseases of the Lung and Windpipe

**Published:** 1837-10

**Authors:** 


### BRITISH AND FOREIGN MEDICAL REVIEW,

FOR OCTOBER, 1837.

### PART FIRST.

### Analytical and Critical Reviews.

### Art. I.

1. *A Treatise on the Diagnosis and Treatment of Diseases of the Chest.* Part I. *Diseases of the Lung and Windpipe.* By William Stokes, m.d., m.r.i.a., Physician to the Meath Hospital, &c.—*Dublin*, 1837. 8vo. pp. 557.

2. *Notes et Additions au Traité de l'Auscultation Médiate de* Laennec. Par M. Meriadec Laennec, d.m.p. &c., et M. Andral, Professeur à la Faculté de Médecine de Paris, Médecin de l'Hôpital de la Charité, &c.
*Notes and Additions to the Treatise on Mediate Auscultation of* Laennec. By M. Meriadec Laennec, m.d. Paris, &c.; and M. Andral, Professor of the Faculty of Medicine of Paris; Physician to the Hospital of La Charité, &c.—8vo. pp. 510; *with two coloured Plates. Paris*, 1836.

3. *Observations on the Surgical Pathology of the Larynx and Trachea, &c.* By Wm. Henry Porter, a.m., Vice-President and Professor of the Theory and Practice of Surgery in the Royal College of Surgeons in Ireland; Surgeon to the Meath Hospital, &c.—*Dublin*, 1837. 8vo. pp. 280.

4. *A Treatise on the Diseases and Injuries of the Larynx and Trachea, &c.* By Frederick Ryland, Surgeon to the Town Infirmary, Birmingham.—*London*, 1837. 8vo. pp. 338; *with Plates.*

We are sanguine enough to believe that medicine is at length really beginning to assume the character of an inductive science. The methods of careful and extended observation and cautious generalization that have of late years so much improved surgery, and elevated it from a state of the blindest empiricism to that of a rational art, have now, in consequence of the multiplied researches in morbid anatomy, and the improved means of physical as well as physiological investigation, become applicable to the study of many internal diseases. In proportion as these modes of study are applied, our facts become more numerous and less fallacious, and our inductions from them more sure; so also our knowledge of diseases increases, and our treatment of them is rendered more successful. But these methods of observation are not to be discovered, nor, when discovered, are they to be exercised, without much labour and skill; and it may be long before they are made profitable in practice. They have, therefore, been pursued by only a few. They do not commend themselves to the indolent or the sordid; and the routine practitioner, who is all alive to the empirical discovery of a new remedy, which may at once become to him a source of profit, neglects, and even dares to ridicule, the researches of a Harvey or of a Hunter, because they do not carry him at once to some easy rule of practice or some wonder-working remedy.

The tardiness with which the study of morbid anatomy advanced in this country, and the obloquy thrown on the important discoveries of Laennec, by which this study has been connected with living pathology, are humiliating proofs of this sordid and indolent spirit among us; and the too-frequent award of public favour, rather to those who cunningly humour the feelings and whims of society, than to those who honestly strive to gain a scientific knowledge of their art, has fostered empiricism and ignorance in every rank of the profession, and has greatly retarded the progress of the science. It is thus that the improvement of medicine will greatly depend on the intelligence and discrimination of the public at large; and a chief reason why we now begin to hope for better things is, that this intelligence is on the rapid increase. The general diffusion of useful knowledge has conferred on every well-educated man some competency to judge of professional merit; and, although the aggregate weaknesses of human nature will always show themselves in the occasional triumph of fashion and caprice over reason and good sense, yet we see around us sufficient proofs that public fame and favour are more and more won by well-exerted talents and scientific industry.

The authors of the works before us, and the works themselves, illustrate the foregoing remarks more fully than we now have time to explain; but there is a circumstance regarding two of these authors which we think it right to notice, because it points out an element almost essential to success, yet not sufficiently appreciated or brought into operation in this country. M. Andral and Dr. Stokes, after an education which comprised the best part of those aids which modern science has conferred on medicine, were enabled to carry their knowledge and talents, with all the undaunted energies of youth, to the bedside of the sick, and there to render them available in extensively observing and interpreting the phenomena of disease. The one, in an unlimited access to the patients in the wards of M. Lerminier at La Charité, the other, as physician to a large hospital at Dublin, found a rich field open to them, and they have cultivated it with assiduity and ability. The valuable productions of these authors are plainly the result of the early devotion of their whole time and talents to the improvement of these advantages. The "Clinique Médicale," a work unrivalled of its kind, and the philosophical "Précis d'Anatomie Pathologique," which is in great measure a generalization of the facts contained in the former work, would probably have never appeared but for this wide field of observation given to M. Andral thus early, when he could devote his whole energies to cultivate it; for, although he has since had extensive hospitals under his own charge, yet he has produced no late work equal to his first; and it is questionable whether, with the multiplied engagements and demands on his time, it be possible for him to achieve such another work as the Clinique Médicale. The many excellent clinical reports already published by Dr. Stokes, and the valuable practical treatise which we have now to present to our readers, afford further proofs of the advantages accruing to science from early placing zealous young men of talent and education in a good field of extensive observation and experience. Had such opportunities, in the case of the authors in question, been withheld even for a few years, the result would probably have been what we continually see in the appointments to great hospitals in this country. The most competent men have to wait in "hope deferred," year after year, until at last, when, haply, they do gain the object of their wishes, their ardour is gone,—much of their knowledge and habit of study lost through disuse,—their minds contracted by the limits of some petty and imperfect sphere of observation to which their circumstances have confined them,—and, last not least, the *res angusta domi* has rendered the engagements of a scanty private practice, or of any other more *direct* way of getting a livelihood, of more pressing importance than working for the science or for the ultimate improvement of the art of medicine. It is a common but great error to suppose that it is always to the advantage of the patients or pupils of a hospital to be under the care of a physician of "some standing;" for, if many of his accumulating years have passed him *standing* without experience, they have left him behind the advance of knowledge; and, if he have succeeded in gaining experience in private practice, his engagements in this will prevent him from giving his time and energies to the duties of the hospital; a great share of which will consequently be left to the subordinate officers. For these, and other reasons also, we would desire to see the appointments to large hospitals limited to a term of years, (say eight or ten;) and, in order that, by this plan, the institutions might not be deprived of the aid of more experienced men, on arriving at the termination of their appointment, the physicians and surgeons might be constituted consulting officers for the same length of time. Without dilating on the advantages of such a regulation, we would express our conviction that it would open the field of scientific practice to a greater number of competent labourers, and it would almost ensure from each of these a greater exertion of talent and industry.

In the present article we propose to review the "Notes and Additions" of MM. Andral and Meriadec Laennec, together with those parts of Dr. Stokes's work which relate to diseases of the chest, reserving for a future Number the section which treats of Diseases of the Larynx and Trachea, and the very valuable monographs of Messrs. Porter and Ryland on the same subjects.

Laennec's discovery of auscultation as a method of diagnosis constituted a small part of his merit. He not only gave us the key to knowledge, but unlocked and set forth its treasures. His fine sense and uncommon talent for observation, joined to a very extensive knowledge of morbid anatomy, enabled him to obtain from his discovery a greater mass of new and important results than that which the combined labours of all his followers have yielded since; so that his last work still stands as the chief text on the subject, to which the additions and modifications of subsequent writers, even the most distinguished, may be appended as notes.* It cannot be denied that many of Laennec's statements have been judiciously qualified by later observers; but one reason for so qualifying them is, not that they were erroneous, but that they were founded on his unrivalled tact and experience in auscultation, and therefore could not be confirmed by ordinary observers; and we truly believe that the confidence which he is said to have unduly placed in auscultation, proceeded as much from his extraordinary and uncommunicable skill in practising it, as from his sanguine partiality to it as its discoverer.

The work of Dr. Stokes being the more complete treatise of those before us, although even he does not profess to include the whole subject or the matter of other works, we may conveniently follow its arrangement, and introduce the notes of M. Andral and M. Meriadec Laennec as occasions occur.

Dr. Stokes divides his book into nine sections, as follows:—Sect. I. General Principles of the Diagnosis of Thoracic Disease; Sect. II. Diseases of the Mucous Membrane; Sect. III. Diseases of the Larynx and Trachea; Sect. IV. Pneumonia; Sect. V. Gangrene of the Lung; Sect. VI. Perforating Abscess of the Lung; Sect. VII. Cancer of the Lung; Sect. VIII. Tubercle of the Lung; Sect. IX. Diseases of the Pleura. There are certain parts of this arrangement which we cannot consider quite consistent with the present state of our knowledge; for instance, the including under diseases of the *mucous* membrane dilatation of the tubes, the pulmonary emphysema of Laennec, and atrophy of the lung; while gangrene of the lung is, with even less reason, separated from pneumonia. Spasmodic asthma and hemorrhage of the lungs are wholly omitted. But these defects, although not unimportant, are much outweighed by the great mass of valuable matter, which, for the most part, is set forth in clear, simple language, and further rendered distinct by summary recapitulations of the conclusions arrived at. His prefatory remarks on the general principles of the diagnosis of thoracic disease are in most instances sound and judicious.

"In the composition of this work, I have kept two great objects steadily in view. Of these, the first is the close connexion of the study of physical signs with that of symptoms, so as to illustrate their mutual bearing on diagnosis, and remove that unjust opprobrium thrown on the advocates of auscultation, that they neglect the study of symptoms. In the next place, I have endeavoured to simplify the subject as much as possible." . . . "I have endeavoured to adapt this work to the wants of the practical man, always assuming that he is familiar with the groundworks of the subject, with the characters and causes of physical signs, as originally taught by Laennec, and more recently investigated in the works of Forbes, Williams, and Clark. Hence I have not entered at any length into the character of physical signs, but rather into the art of reasoning justly upon them; for it is in this that most observers fail. It cannot be too often repeated, that physical signs only reveal mechanical conditions, which may proceed from the most different causes; and that the latter are to be determined by a process of reasoning on their connexion and succession, on their relation to time, and *their association with symptoms.* It is in this that the medical mind is seen. Without this power, I have no hesitation in saying that

* Nothing can better show the permanent estimation in which the work of Laennec is held, than the fact that some other works on Diseases of the Chest, which have obtained considerable circulation, are almost a transcript from it, with very little additional matter.

it would be safer to wholly neglect the physical signs, and to trust in practice to symptoms alone." (*Preface*, p. ix.)

This opinion should, we think, be qualified by the reflection that, without this "power," or "process of reasoning," *symptoms* also are but blind guides, and often tend to mislead as much as unintelligible physical signs. The following remark shows more strongly and truly the value of physical signs.

"As far as was possible, I have shown the utility of physical signs in practice; for it is in the curable diseases that their great value is seen. Indeed, in a large proportion of such cases, the first effect of treatment is to render disease latent, and to cause an absolute necessity for the study of physical signs." (P. xi.)

Dr. Stokes observes, that the labours of modern pathologists in the localization of disease have done much to diminish the uncertainty of medicine; and this chiefly by increasing our knowledge of the signs and symptoms of disease. The word *signs* Dr. Stokes restricts, after the manner of Laennec, to those which are physical, recognizable by the senses, and depending on physical alterations in the conditions and relations of parts; whilst *symptoms* proceed from changes in the functions or vital relations of the suffering organs. This is an arbitrary application of the terms, but it may be a convenient one. The word symptom properly signifies a coincident; and, although perceptible changes of function and vital relation are often more certain effects of disease than this would imply, yet their inferiority, in point of connexion with the pathological condition of an organ, to the physical signs, which are an essential part of that condition, may be somewhat represented by this distinction of terms. Symptoms may, then, be considered in a threefold manner:—1st. Changes in the functions of the part itself; 2d. Changes in the phenomena of organic life in various parts of the system; 3d. Changes in the phenomena of animal life. The first is a separation of the primary local from the general phenomena; for the latter two, which comprehend another division into organic animal, would otherwise include the first.

"Thus, in examining the symptoms of a disease of a particular organ, we investigate the state of its own functions. We then examine the changes caused by disease in all the phenomena of organic life, such as digestion, respiration, circulation, absorption, nutrition, exhalation, secretion, and animal heat. From these we advance to the phenomena of the life of relation, and examine the changes produced in the muscular power or function, the organs of sense, the moral affections, and the intellectual manifestations. In a case of acute inflammation of the lung, we observe, in the first place, lesions of its own function, painful and hurried respiration, imperfect arterialization of the blood, cough, and expectoration: these are what may be called local symptoms; but we may have others referrible to the disturbance of organic life in parts distinct from the lung: thus, we observe excitement of the heart, fever, and various derangements of the digestive and urinary systems: further, in certain cases there may be signs of a lesion of the phenomena of the life of relation; as, for instance, prostration of strength, and other signs of derangement of the cerebro-spinal system. It must be obvious that, in the detection of the nature and seat of any disease, the more we can combine the observation of physical signs with functional symptoms, the greater will be the accuracy of our diagnosis. Now, if we compare together the diseases of the three great splanchnic cavities, we find that those in which this desirable combination is most attainable are, first, those of the chest; next, the abdomen; and, lastly, the affections of the brain and spinal marrow. Accordingly, if we compare the diseases of these systems with respect to the perfection of diagnosis, we find the order to be, first, the respiratory; next, the abdominal; and last, the cerebro-spinal, or that in which this combination is least applicable." (P. 3.)

Dr. Stokes's reasons why the viscera of the chest are, above all others, calculated to give physical signs of their condition, will admit of compression.

1. The chest contains air in certain quantity, which quantity is changed by disease, and can be appreciated by percussion.

2. The division of the chest into two halves, and to some degree of each lung into lobes, circumscribes the physical changes to limited parts, and gives a facility of detecting them by comparison with other parts.

3. The regular and notable motions of the pectoral organs furnish another element of diagnosis between their healthy and diseased condition.

4. The phenomena of the voice extend to the contents of the chest, and may be modified by disease there.

5. The shape of the chest may be so changed by disease of the organs within as to give signs of the existence of that disease.

6. The air in every part of the lungs being in continual motion occasions sounds which become signs of the condition of the structure of each part. (This might be included under the heads 1 and 3.)

7. The mobility of the organs, and the yielding nature of parts of the walls of the chest, give rise to various displacements, which may afford physical signs. Each of these heads is fully explained and illustrated. Thus, in speaking of the fifth source of signs, change of shape, we have the following remarks:

"If we look to the affections of the head and spinal cavity, we find that, with the exception of some few cases of congenital dropsy, arrest of development, or chronic effusion, the more frequent diseases of this class do not produce any perceptible alteration or change of shape in the bony cases of the cerebro-spinal mass. In the abdomen, on the other hand, from the very yielding nature of its parietes, changes of volume and shape are common; but it will be found that these seldom are available for the detection of the nature of their cause; a circumstance as well attributable to the great yielding of the parietes, as to the fact of the viscera being contained in a single cavity. The chest, however, presents bony, elastic, and fleshy parietes, and its principal viscera occupy three distinct cavities.

"Although it cannot be maintained that the alterations of shape and volume of the chest will always suffice to point out the nature of their causes, yet we must admit that, with respect to the diseases of its interior, the modifications of its exterior are more numerous and of greater diagnostic value in the chest than in either of the other two cavities. Let us consider the extraordinary convexity of the whole chest, the arching of the sternum, and the appearance of the shoulders, in a case of dilatation of the air-cells; the loss of symmetry in the sides, and the peculiar smooth appearance produced by the pressure of the fluid on the intercostal spaces, in the case of empyema; the contraction of the side and the depression of the shoulder while the spine remains unbent, in the same case, where absorption of the fluid has taken place; and the sunken and flattened appearance of the antero-superior regions in advanced phthisis. All these are instances of peculiar modifications of shape of the exterior walls of the cavity, coinciding with physical changes in the subjacent viscera. It is true that, taken alone, they could not lead to a positive diagnosis, but, when combined with other signs and symptoms, their value is highly important; and this, with their number, should make us admit that, as a means of diagnosis, the modifications of external form produced by disease are more valuable and much more frequently applicable in thoracic than in the cerebro-spinal or abdominal affections." (P. 7.)

We think that Dr. Stokes might have given another comprehensive reason why the diseases of the chest may, better than those of other cavities, be interpreted through physical signs. The structure and functions of the thoracic viscera are better understood and more mechanical than those of the head or abdomen. The organs of respiration and circulation exhibit a mechanism which is admirably adapted to the accomplishment of the known objects, the aeration and circulation of the blood. The working of this machinery must necessarily be attended with notions appreciable to our senses, and thus constituting the elements of physical signs. In the abdomen, the functions of the viscera are complicated with the more obscure vital properties of secretion and digestion, and therefore less fitted to be represented by physical signs; and in the brain and spinal marrow there is no intelligible working of machinery; there is therefore the lowest capacity to yield physical signs, and functional symptoms must be the only interpreter.

Laennec well remarked, and Dr. Stokes enlarges on the remark, that without physical signs thoracic diseases are often more difficult of detection than those of other cavities. Still our author does most wisely to insist on the absolute necessity of attending to the general as well as to the physical signs. He truly remarks that "great injury has been done to the cause of physical diagnosis by some inexperienced men who, departing from the principles of its illustrious founder, have neglected too much the study of symptoms."

The discursive style which Dr. Stokes adopts renders him at times somewhat prolix; and, although there is everywhere the stamp of good judgment and sound experience in his illustrations, the want of a more concise and simple arrangement exposes him to occasional repetitions. Thus, in enumerating the sources of physical signs, there is necessarily a repetition of what had been said in describing the aptitude of the chest to yield physical signs; and under the heads "mutual dependence of signs and symptoms," "insufficiency of signs alone," and "relation of signs to symptoms," we have the same matters again and again brought under our notice. This repetition may perhaps be useful in impressing the more important subjects on the mind of the reader; and the following quotations will show the weight of the positions and value of the illustrations adduced.

"It is never to be forgotten that, although in these various classes, we have a vast number of well-marked and essentially differing physical phenomena, *there is not one of them, which, taken singly, can be considered as a pathognomonic sign. Nay, we might go further, and declare that no possible combination of them can be considered absolutely pathognomonic.* By some of them, taken singly, or by various possible combinations, we may indeed ascertain the existence of certain mechanical conditions of the intra-thoracic viscera, as, for instance, permeability or impermeability, increase or diminution of the quantity of air, the existence of cavities of various sizes and with various communications, the roughened state of a serous membrane, or the displacement of particular organs; but, if we seek to determine by physical signs alone, the cause of all or any of these phenomena, we shall find it to be difficult or impossible. It is only, as we have said before, by the connexion of the accurately ascertained physical signs with the previous history and actual symptoms of the case, that a correct diagnosis can ever be arrived at. In order to establish the proposition that no physical sign, taken singly, can be considered as pathognomonic, let us take a brief review of these different signs, commencing with those least frequently applicable, and proceeding to those of most common occurrence.

"1st. *Existence of an external collateral venous circulation.* This appearance, which has been described by Reynaud, is indicative of a great amount of obstruction to the internal venous circulation. But of the nature of that obstruction it alone can tell nothing. It may proceed from the presence of a tumour, aneurismal or otherwise, or from disease on the internal surface of the venous trunk itself. This was observed to occur in the vena portæ and inferior cava in a patient whose case is described by the same author, and in whom the superficial veins of the abdomen took on a supplementary action. In obstructions of the right side of the heart, the dilatations of the jugular veins so long noticed seems to be a commencement of the same morbid appearance, and Dr. Graves has shewn that a varicose state of the superficial thoracic veins may oocur from cancerous degeneration of the lung itself. If, for the sake of argument, we assume that these different causes for the appearance in question were of equal frequency, and that from it alone we determined on the existence of any one of them, there would be four chances to one against our making a correct diagnosis.

"2d. *Signs derived from the displacement of the thoracic or abdominal viscera.* Of these, those that are most frequently recognized are the displacements of the heart and liver; the first is commonly observed in cases of empyema, the displacement to the right of the mesian line occurring in empyema of the left side, while that in the opposite direction indicates accumulation in the right pleura. Now, although displacement of the heart to the right side of the sternum constitutes one of the best indications of empyema of the left pleura, yet, taken alone, it is anything but unequivocal. A tumour or an hypertrophy of the left lung may produce a pulsation to the right of the sternum; the same may be caused by an hypertrophy and dilatation of the right cavities of the heart: and Dr. Graves and I have shewn that an aneurism of the aorta may push the heart to the right side. I have also published the particulars of an extraordinary case of dislocation of the heart from external violence, in which the organ was drawn far to the right of the mesian line, and in which no sign of empyema of the left pleura had ever occurred. When I come to treat of the affections of the heart I shall give the particulars of this case. Lastly, well-attested examples of congenital displacement of the viscera have been recorded, in which the heart was placed at the right of the mesian line. On the other hand, displacement of the heart towards the left axillary region is a circumstance which, from its nature, is commonly overlooked, and which may occur from other causes. I may also remark, that the previous contraction of either side from a former attack of pleurisy should be added to the possible uncertainties of this source of diagnosis, for in such cases, the heart seldom resumes its normal situation with respect to the healthy side.

"As the displacement of the heart, considered alone, and without reference to any acoustic observation, is reducible, as a sign, to the mere feeling or seeing its pulsations in an anormal situation; so the displacement of the liver is reducible to the observation of a tumour in the right hypochondrium. Now, even supposing that the case was one of displacement of the liver, it will be shewn that this might arise from other causes than empyema, to which it is commonly attributed; intra-thoracic tumours might produce it. I have observed it from Laennec's empyema; it may occur from aneurism of the abdominal aorta, or from that of the hepatic artery; and I need scarcely remark, that we may have hepatic tumors, independent of any disease of the pleura; and, conversely, pleural effusion without this sign. These observations are sufficient to shew that displacements of the heart or liver cannot alone be looked upon as certain diagnostics of the lesion which has produced them.

"3d. *Signs derived from the inspection of the motions of the tharax during respiration.* * * * The respiratory movements are so infinitely various in the different diseases of the chest, that we are not warranted in founding any certain diagnosis upon the observation of them alone.

"4th. *Signs referrible to the sense of touch.* This class presents to us several signs which, as far as they go, lead to a greater degree of certainty than those in the preceding one. Yet, like the other physical signs, they only reveal to us, and that not constantly, mechanical conditions, without leading to the diagnosis of the nature of disease, or the pathological state of the viscera. Thus, the bronchial vibration may occur from any liquid effusion into the tubes, and with various states of the lungs. The feeling of gurgling may proceed from a tuberculous, pneumonic, or gangrenous abscess, or from a dilated tube containing muco-puriform matter. The cause of the sensation of friction has not been sufficiently investigated, but we know that the rubbing feel may arise in various states of the serous membranes, while that of non-expansion of parts of the lung will obviously be produced by many different causes. The cases in which the sense of touch leads us to most certainty in diagnosis, are those of the diseases of the heart and great vessels; yet every practical man knows that the most violent impulses occur without organic disease of the circulating system, while, on the other hand, extensive hypertrophy of the heart may exist with a natural impulse, and an aneurism of the aorta give no morbid pulsation.

"5th. *Signs derived from alteration in the shape and volume of the thorax.* In this class of signs we meet with some of considerable value; thus, the convexity of the chest in Laennec's emphysema, when carried to a great degree, is an appearance almost peculiar to the disease; and which, combined with the elevated shoulders and the hypertrophied state of the muscles in the neck, will scarcely mislead. But, of the various partial dilatations and contractions, there is no one at all pathognomonic: many of them may be congenital, or the result of former and of various diseases. Thus, dilatation of either side may arise from emphysema, pneumothorax, pleural effusions of various kinds, effusions into the pericardium, enlargements of the liver, or aneurisms of the aorta. An apparent dilatation, too, may exist, in consequence of the contraction of the opposite side, and contraction itself may arise from a variety of morbid causes or be a congenital conformation.

"6th. *Signs referrible to acoustics.* These have been hitherto divided into those obtained by *percussion,* and by *mediate* or *immediate auscultation*; a division which seems to be unnecessary, as both classes of signs, being appreciable by the ear alone, should be ranged under the general head of *auscultatory phenomena.* Under this head, therefore, we shall treat of Percussion, and Auscultation, whether Mediate or Immediate. Previous, however, to our entering on an investigation of their value as diagnostic means, we shall briefly describe the principles of these modes of diagnosis. It is plain that we have acoustic phenomena referrible to a passive and an active state of the lung; in other words, to conditions, on the one hand, independent of motion or life; and, on the other, inseparable from them. The passive phenomena, or those of percussion, which relate merely to the quantity of air within the thorax, may be as well observed in the dead as in the living body; while the active, or those of respiration, the voice or the phenomena of the heart and arteries, imply motion and life. Hence we may divide the phenomena of auscultation into those of the passive and active conditions." (P. 15-19.)

In comprehending *percussion* under the head *auscultation,* Dr. Stokes has been anticipated by Dr. Forbes, who, in the Cyclopædia of Practical Medicine, includes all these phenomena in the article "Auscultation." The division of the phenomena into *active* and *passive* may be convenient, but we do not think the expressions logical. If an innovation be desired, the words statical and dynamic, applied by philosophers to forces at rest, and forces in motion, would be more correct. But we question the expediency of changing the terms in common use: they correctly imply the mode of obtaining the phenomena, which is of more importance than whether the phenomena result from natural or from artificial movements in the chest.

In practising mediate percussion, Dr. Stokes uses the finger with its *back* turned to the chest, as a pleximeter. The fingers are now generally preferred to an instrument. The invention of this convenient mode of mediate percussion is ascribed by Dr. Edwin Harrison to Dr. Skerrett, who used it at the time of M. Piorry's early researches.* M. Andral, also, says he constantly employs this method; and he remarks that, although a dress of linen or of flannel on the patient's chest does not

* Med. Gazette, Feb. 18, 1837.

interfere with the sounds elicited by percussion, some other tissues, as cambric muslin, do so in a singular degree. We presume that this must be when they are either too much stiffened or not in close contact with the walls of the chest.

In investigating the principles of percussion as a mode of obtaining signs, Dr. Stokes states that "the sound on percussion is directly as the quantity of air contained within the thorax." But, in exemplifying the phenomena, he finds it necessary to make the sound depend on the quantity of air in the *part* of the thorax where the percussion is applied; and thus the principle of *comparison* becomes an important guide in the practice of percussion. This is an approach to the view of Dr. Williams, which we brought before our readers in our Number for April. This view is that the sound of percussion is not seated in the air, but in the solids, and that, on percussing the chest, its walls will vibrate and yield a sound according to the nature of the substance which is under them. If that substance be light and elastic, as healthy lung, the vibrations will be free and the sound full; if it be more solid or liquid, the vibrations will be resisted or choked, and the sound dull. If this view be correct, (and it certainly seems to be more consistent with all the phenomena of percussion, as well as with the laws of acoustics,) the sound is not in proportion to the quantity of air within the chest, but in proportion as that air is placed at the surface of the lung, and permits the free vibration of the walls of the chest. Thus, in bronchitis and some cases of phthisis and pneumonia, although the air in the chest is much diminished, the sound on percussion is very little, if at all impaired, because in the superficial parts of the pulmonary tissue, there is as much air as usual, or it may even be increased by a superficial emphysema. Dr. Williams has suggested the means, by varying the *force* of percussion, of discerning between these *surface*-sounds and those of the deeper-seated parts.

In a note on the sounds obtained by percussion of different regions, M. Andral remarks that Laennec had omitted to notice the dulness produced by the heart in the left submammary region. This, according to the measurements of M. Piorry and Bouillaud, amounts to one and a half or two square inches, which represents that part of the heart not covered with the lung. This dulness is by no means a measure of the size of the heart, as this organ, acquiring a large volume underneath, a well expanded or emphysematous lung might cause no increased dulness on percussion, unless indeed by the methods of modified percussion proposed by Dr. Williams. M. Andral thinks that dilatation of the air-cells of that part of the lung which overlaps the heart, frequently disguises the results of percussion by yielding an unusually clear sound. In our own experience, we have found the tympanitic resonance of the adjoining stomach a much more common source of perplexity. Neither Laennec nor Andral sufficiently notices the manner in which the *liver* impairs the sound of the lower parts of the chest. This omission has been pointed out by Dr. Harrison,* who justly remarks that the liver often diminishes, although it does not destroy, the pectoral resonance as high as the fourth rib on the right side, and on the left the dulness of this organ may be mistaken for that of the heart.

* Med. Gazette, Feb. 18, p. 776.

With regard to the stethoscope, Dr. Stokes merely recommends "that it shall consist of but one piece, be constructed of cedar or some light wood, have its bell small and with rounded edges, and the ear-piece sufficiently concave." From this notice, and from the light estimation in which Dr. Stokes holds the signs produced by the voice, we presume that he never makes use of a stopper to the instrument. This is neglected very much in this country also, and we believe, very unwisely; for this little appendage furnishes the means of testing some signs that are otherwise very equivocal. We may here notice M. Andral's note on the statement of Laennec, that those "who confine themselves to the *immediate* mode of auscultation will never acquire a great certainty in diagnosis." M. A. does little more than deny that he has experienced any of the imperfections and difficulties which Laennec ascribes to immediate auscultation. On the contrary, he has found it more easy to both physician and patient, and as distinct in its results: the objection against this mode, on account of the perspiration or filth of patients, can always be removed by interposing a handkerchief or napkin; and the noises of friction of the head with the clothes, &c. can always be guarded against by using steady pressure. He admits certain cases of malformation, the examination of sounds of the arteries, and the phenomenon pectoriloquy to be the only instances in which the stethoscope is better than the unarmed ear, and for these instances it may be used. We have had a great deal of experience in both methods of auscultation; and we think that, although Laennec, in his zeal for his instrument, has exaggerated the objections to immediate auscultation, he has by no means overrated the advantages of the mediate method. There are very many patients beneath whose clavicles the ear cannot be closely applied; and yet how important is this region for examination! Again, we must add our experience to that of Laennec against M. Andral, and maintain that the stethoscope separates far better than the simple ear the sounds produced in particular spots. This circumstance gives it a great advantage in determining the nature of a vocal resonance, a bronchial respiration, and the precise situation of the various sounds of the heart. If to these cases we add those in which the stethoscope becomes preferable on the score of delicacy and cleanliness, there will be reason enough to induce us to carry the stethoscope always with us. It is, beyond doubt, more difficult to learn mediate auscultation than immediate; just as it is easier to whistle than to play the flute. The stethoscope requires considerable practice, and, to gain this practice, we would recommend students to use the instrument at first as much as possible in *all* cases, and they will then soon be masters of it in those cases where the naked ear *cannot* be applied. This once gained, they will soon learn to abridge their labour where the easier method can be used, without injuring their powers for nicer points.

Dr. Stokes gives very clearly the principle of the *active* auscultatory phenomena.

"The manner in which the stethoscope assists us in detecting the state of the thoracic viscera can be explained in a very few words. The air, as it passes through the lungs in the acts of inspiration and expiration; the sound of the voice in different parts of the chest; and the impulse and sound of the heart at each pulsation, have all certain characters in the state of health. They present phenomena which are to be considered as standards of comparison. Now, every disease of the lungs and heart alters or modifies these characters, according as the case may be; and it is by the knowledge of the *morbid phenomena* or deviations from the natural state, that we may judge of the state of the thoracic viscera." (P. 23.)

The following principles are announced by Dr. Stokes as governing the application of physical signs to the detection of disease:

"First. That the value of most of the preceding signs, or of their combinations, in the determination of the seat, nature, or extent of disease, is to be estimated more by comparison with the phenomena of other portions of the chest, than by their mere existence in a particular situation.

"Second. That the greater the number of physical signs which can be combined in any particular case, the more accurate will our conclusions be. But, of these combinations, the most important and indispensable is that of the passive and active auscultatory phenomena.

"Third. That the existing physical signs are to be considered in relation to the period of duration of the disease, and the rapidity or slowness of their own changes.

"Fourth. That, in all cases, the value of physical signs must be tested by the existing symptoms and previous history; while, on the other hand, the observation of these physical signs enables us to correct the conclusions to which we would be led by the unaided study of symptoms.

"I shall proceed to the elucidation of the principle of COMPARISON. This principle, which may be said to be the basis of physical diagnosis, has not been sufficiently insisted on, either in the work of Laennec or of any of the succeeding writers on auscultation. Indeed, Dr. Williams is the only author who alludes to the subject: but even this author does not sufficiently insist on its paramount importance, and refers to it principally as connected with the use of percussion." (P. 24.)

We do not think that Dr. Stokes does justice on this point to the authors that have preceded him. It is true that they have not, as he has, laid down *comparison* as an abstract principle, to be applied to *all* the means of physical diagnosis; nor are we sure that its application ought to be so special and universal as our author insists. There are many signs which are in themselves positive, without the aid of comparison: thus, the rhonchi, the sound of friction, and metallic tinkling, often need no comparison with other parts of the lung to determine their existence; the examination of other parts may or may not give additional information, but it is a misapplication of the term to make the word comparison imply a perfect examination. It is quite true that in this, as in every case where the judgment is exercised, there must be comparison; but in this sense comparison is equally necessary in studying general symptoms, and, in fact, in every pursuit of inductive science. The special applicability of comparison to auscultation is founded on the slight differences between the signs of health and of disease, or of diseases of different kinds. These signs not being sufficiently distinct in their positive characters, may become so on being closely compared with healthy signs in the same subject. Thus, the early signs of phthisis, feeble or slightly bronchial respiration, bronchophony, and dulness on percussion, are not sufficiently positive to declare themselves morbid until they are compared with the phenomena of parts that represent the more healthy standard of the same subject. In this and in similar cases, we find Laennec adverting to the signs being most evident on one side. Dr. Forbes, in his article on Auscultation before quoted, particularly alludes to the greater value of signs when they occur partially, "particularly if on one side only." Dr. Clark, in his treatise on Pulmonary Consumption, frequently speaks of dissimilarity between the phenomena of the two sides, as furnishing the truest signs of the existence of disease; and Dr. Williams, besides, in the passage quoted by the author, insists again and again that these signs are most certain " when they are more manifest on one side than on the other," and " when there is a distinct difference between the two sides of the chest;" and, in speaking of the mode of auscultation, recommends the observer not to change sides, but to lean across, " for the comparison of corresponding points on both sides, where it is important that the two impressions should succeed quickly to each other." Similar quotations might be made from other writers, to show that this principle has been by no means neglected, although Dr. Stokes now upholds, and applies it more ably and fully than the authors who have preceded him. The following illustration is one in which he is original.

"Comparison must be used in determining the value of the modifications of the original active phenomena, as well as of that of the new or non-analogous signs. A good example of this is seen in the detection of foreign bodies in the bronchial tubes; for it is principally by the comparison of the respiratory sounds in both lungs that the diagnosis of a foreign body can be arrived at. To this subject I shall return hereafter. I may also observe that, in certain cases of aneurism of the aorta or innominata, it is by a comparison of the respiratory murmur in either lung that the existence of a tumour at an early period can be detected."

"One of the most striking instances of the difficulties which arise when the application of comparison is fallacious, is that of the development of tubercle in a patient whose chest has been deformed from previous disease. Patients who have recovered from empyema with a contracted side are liable to tubercular development, and the stethoscopist may be called to determine the question as to whether tubercle exist or not. I have been more than once in this situation, and believe that a more difficult case for diagnosis can hardly be met with. The symptoms will seldom afford any assistance, as they may proceed either from incipient phthisis or be those commonly present during the convalescence from empyema; and, in consequence of the previous disease of one pleura, and the contraction of the chest, we are deprived of the advantages of comparison of the phenomena of both lungs by the stethoscope and percussion. Thus, if we find the side originally affected to be duller than the other on percussion, this may be explained either by the diminished volume of the lung or by the development of tubercles. The same difficulty exists in the observation of respiration and the phenomena of the voice." (P. 27-8.)

The importance of a due combination of the signs, particularly those drawn from percussion and the stethoscope, which was strongly insisted on by Laennec, is happily exemplified by Mr. Stokes. He also makes some very judicious remarks on the consideration of signs in reference to time, and the changes which it induces. On this principle, in great degree, hangs the diagnosis of dilated bronchi, foreign bodies in the trachea, acute general development of tubercle, emphysema of the lung, certain cases of empyema and pneumothorax, hydrothorax, nervous palpitation of the heart, pericarditis with effusion, rupture of an hepatic abscess into the lung, and sympathetic cough. Dr. Stokes treats this subject with the practised hand of one whose views have been enlarged by an extensive familiarity with clinical practice. The value of his illustrations tempts us to make extracts from both this and the last head of this subject, in which he proves the necessity of correcting mutually the physical signs and the general symptoms by each other, but our limits will not permit, and we must refer to the work itself.

The "Notes to Laennec," on the general Phenomena of Auscultation, comprise little more than *résumés* of the signs by M. Meriadec Laennec. They may be useful to his countrymen, as few seem to take the trouble to comprehend fully the text of the great original: in England they are unnecessary, as we have several excellent summaries in original works. Of the additions of M. Andral, there is one of some importance on the relation of the different rhonchi to the acts of inspiration and expiration. He states that the true crepitant rhonchus, which he calls, from its supposed seat, *râle vesiculaire,* is heard only during inspiration. The mucous and submucous rhonchi, which sometimes otherwise resemble the crepitant, accompany both inspiration and expiration, or the latter only; and the same remark applies to the sibilant and sonorous rhonchi, which also have their seat in the bronchial ramifications. From our own observation, we think that the crepitant rhonchus in the early stage of peripneumony, accompanies expiration as well as inspiration; and it is only as the obstruction becomes more complete that it is confined to the inspiratory act. Now, as much the same thing occurs with the finer kinds of mucous rhonchus, we doubt that this can be made a ground of distinction. M. Andral states that he has verified the sign of the rubbing sound between the pleuræ, predicted by Laennec, and first observed by M. Reynaud. In the case of a young man recovering from a pleuritic effusion on the left side, it continued for three months, and was felt by the individual long after he was restored to health.

The next section, on Bronchitis, is the most voluminous and important in Dr. Stokes's work, and contains a great mass of valuable matter. It includes, however, more subjects than are generally considered to belong to it, and it appears to be a prominent aim of the author to make the study of bronchitis a key to thoracic pathology. Thus, we find comprehended under the head of bronchitis, not only the positively inflammatory diseases of the bronchi, but every kind of disorder, functional and organic, which affects the bronchi to their vesicular terminations: nay, we have dilatation and atrophy of these terminations themselves. Dr. Stokes thus places himself, with Broussais, at the farthest extreme from Laennec, who prefers the term catarrh even for the acute affections of the bronchi, not admitting that their inflammatory character is certain. The middle course taken by M. Andral is, we think, the most natural. Considering inflammation as a complex phenomenon, consisting of several processes, he admits that each of these may exist separately, and produce affections distinct from inflammation. Thus, the secretion or the nutrition of the bronchi may be modified without inflammation, although inflammation, when present, essentially tends also to change these processes; and we may have a bronchorhœa or catarrh, or an atrophy or hypertrophy of the bronchi, without any evidence that inflammation has preceded or accompanied it. These distinctions are the more important, because they are associated with marked differences in practice; and we shall find that Dr. Stokes, although he associates all affections of the bronchi with inflammation, recommends stimulant remedies in the apyrexial forms as strenuously as those who drop in the name an hypothetical connexion that ceases to be practically useful. Inflammation must, indeed, be a variable process to produce even the changes of secretion enumerated in the following classification: how much does it differ in extent from that which others would call *disease?*

"In classifying the different forms of bronchitis, we may take for the basis of our division the different immediate results of irritation of mucous membrane and glands. In the first, or most ordinary form, we have a mucous, and afterwards a muco-purulent secretion; in the second, we have a secretion bearing the character of lymph, as in some of the forms of croup; in the third, the secretion is principally serous, as in the different forms of humid catarrh and asthma; whilst, in the fourth, there is little or no secretion, a disease which has received the name of the dry catarrh. It may be remarked that, in certain cases, the more copious and elaborated the secretion, the greater is the relief produced: thus, a mucous expectoration gives more relief than a watery; a muco-purulent more than a mucous; and a purulent, perhaps, more than any." (P. 46.)

The remarks on the bronchitis attending dentition are excellent.

"One of the simplest and mildest forms of this disease occurs about the period of the first dentition, and it seems likely that it is not then a primary disease, but rather the effect of the general constitutional disturbance, as we often observe it arising either along with or subsequent to the irritation of the gums, and subsiding after the adoption of means calculated to relieve these parts. Nor is bronchitis a constant attendant on dentition; for irritation may be localized in the abdomen, the head, or the skin, all which tends to show that the bronchial irritation is not the first link in the chain, and that its occurrence is accidental and secondary. That this doctrine is important in a practical point of view, no one can doubt; yet, whether it may be shown that the bronchitis be the cause or the effect of the fever, the detection of its existence is of importance, and its removal absolutely necessary. There is no difficulty in recognizing this affection, even though it should exist in an apyrexial form. Under such circumstances, the child may be observed to be irritable, his breathing hurried, with a slight wheezing in the throat and acceleration in the pulse. In more severe cases, there is [are] fever and cough, the nares dilate during inspiration, and the act of sucking seems to be performed with difficulty. If we examine the mouth, we often find it hot, and the gums dry and swollen, and one or two teeth may be observed coming forward. I have more than once found that such an attack supervened in children who had had copious dribbling for a length of time previously, and that the arrest of this secretion preceded the bronchitis and constitutional disturbance. In some cases, the cough has a decidedly croupy character, although during the intervals the breathing, though hurried, is not at all stridulous. This character of cough is often a source of great alarm, and may lead to an unnecessary degree of activity in practice. The symptoms, such as have been described, continue from four to five days, and often subside rapidly on the appearance of a tooth, although they may be liable to return upon every new irritation of the gums." (P. 51.)

Dr. Stokes divides cases of bronchitis into the primary, secondary, and complicated forms. The primary is the idiopathic disease; the fever, if any be present, being symptomatic. The secondary is where the inflammation of the lung is consecutive to some other disease, especially fever. The complicated form of bronchitis is where it accompanies other disease of the lung: this is not considered separately. The acute and chronic varieties of primary and secondary bronchitis, with their signs, symptoms, and pathological tendencies, are fully considered in the next fifty pages, which are most deserving of the careful perusal of the student and practitioner. They will scarcely admit of condensation, or we would wish to give our readers some account of them. We must content ourselves with a few extracts; and, if we now and then express our dissent from certain opinions, this must not detract from the high estimation in which we hold this work, which would become a standard one if it only contained the matter of this section.

"But although, in its milder forms, the primary bronchitis is a common affection, yet the more violent attacks of this disease are far from being frequent, at least in those of mature age; for, in the great majority of the cases of acute bronchitis which come before us, we see it either as supervening on some chronic affection of the lung or as a secondary disease, such as that which arises in the course of the eruptive and continued fevers. Indeed, the more violent primary bronchitis, though common in the child, is a rare disease in the adult; while, with respect to the chronic forms of the affection, the reverse seems to be true, as this latter is common in the adult, and comparatively rare in the child. Acute primary bronchitis may terminate by resolution; it may pass into a chronic and increasing flux from the bronchial membrane, with or without hectic, giving rise to various alterations of the lung; it may cause death by a sudden obstruction of a large tube; it may be accompanied by a rapid or followed by a slow development of tubercle; it may pass into pneumonia, or terminate fatally by an excessive secretion into the bronchial tubes, or by hydrothorax." (P. 56.)

The following extract will serve to illustrate the remark we have made on the doubtful inflammatory nature of some affections which are classed under the name bronchitis.

"It is not easy to draw the line of distinction between this affection (chronic primary bronchitis,) and the second stage of the last variety, as we may observe it either as its continuation, with certain modifications, or as an affection in which there never have been the precursory inflammatory symptoms. We may get a good idea of the ordinary form of this disease, by considering it as a species of gleet of the mucous membrane, in which the inflammatory irritation, *if it exists,* is in many cases not so severe as to act sympathetically on the system; so that patients, under these circumstances, although labouring under cough and expectoration, may yet preserve a good state of general health. Nutrition may go on well; there may be no fever whatever, and even but little dyspnœa, unless upon considerable muscular exertion. In such cases, there is generally a more or less complete remission of the symptoms during the summer season, but, when winter approaches, the cough and expectoration become more troublesome, again to subside on the approach of summer. Thus may these patients continue for years, when the duration of the remissions becomes less, their completeness diminishes, and a permanent irritation and flux are established." (P. 57.)

Dr. Stokes divides the secretions from the bronchial mucous membrane, when in a state of "irritation," into transparent mucous secretions; opaque mucous or albuminous secretions, which may be either amorphous or moulded into the form of the tubes; muco-puriform secretions, puriform secretions, and serous secretions. The *transparent mucous secretion* generally occurs in the early stage of acute bronchitis, or when the disease, in any of its forms, undergoes an exacerbation: this has been long since described by Andral and others. The *opaque mucous secretions* generally succeed to the former kind, and may either appear in shapeless masses of a dull white colour, with a slight yellow tinge, more or less mixed with serum, or in plugs or cylinders of a tough white mucus, moulded into the shape of the bronchial tubes. In the case of a patient who suffered under a chronic affection of the chest, "the casts expectorated were several inches in length, and seemed to have occupied the bronchial tubes from about their third order to nearly their finest ramifications." The memoir of M. Reynaud on a plastic kind of inflammation of the minute bronchi, is also referred to. The *muco-*

*puriform* and the *puriform* secretions are properly considered together, for they are but degrees of one morbid condition. Dr. Stokes considers an absolutely puriform secretion to be very rare in any pulmonary disease, but particularly so in bronchitis. Yet we presume many of our readers, as well as ourselves, have known a patient expectorate real pus in considerable quantities, and, on examination after death, not only were the lungs sound, but the bronchial membrane entire, and generally pale throughout. Dr. Stokes says, that the muco-puriform expectoration most commonly takes place in the second stage of acute bronchitis, especially where this has occupied the smaller tubes, and produced a muco-crepitating rhonchus. If the antiphlogistic treatment have not been used early or carried far enough in the first stage, intense bronchitis passes into the second, marked by this muco-purulent secretion, which may be so abundant as to cause suffocation. But the change from mucous to muco-puriform expectoration may be sometimes considered a favorable one, leading to the termination of the inflammation. It may be viewed as such when the symptoms also show an improvement, and when the muco-crepitating rattle becomes larger, the air heard to penetrate lower, and the sound on percussion is clear even in the postero-inferior portions of the lung. The occurrence of purulent matter in the expectoration, with other symptoms, becomes a useful index to point out the time for changing the treatment from antiphlogistic to stimulating. *Serous secretions* occur in many cases of bronchitis, phthisis, and, we would add, other diseases of the lungs and heart. Sometimes they are superadded to some of the preceding kinds of expectoration, and Dr. Stokes seems to think the idiopathic pituitous catarrh of Laennec a rare disease; but, when the pituitous or serous discharge constitutes the chief symptom, which it does in many cases of *humoral asthma*, we do not see why the occasional mixture of a little concocted mucus with it should deprive it of its idiopathic character. In the article *Catarrh*, in the Cyclopædia of Practical Medicine, by Dr. Williams, the combination of pituitous with the dry catarrh of Laennec is adverted to as not uncommon, tough mucus being expectorated with the serous fluid; and, when the one is viewed as a profluvium, and the other as a passive congestion, both may be dependent on want of tone in the bronchial capillaries, and therefore may readily occur in different parts of the same membrane. Whether this view be correct or not, we think that Dr. Stokes would have done well to follow Laennec rather than Broussais in this instance. M. Andral, in his Notes, refers to cases in his Clinique Médicale, illustrating the pituitous catarrh of Laennec, which he considers simply a disease of secretion, although it may occasionally arise from and terminate in a hyperæmia.

The physical signs of bronchitis are fully described by Dr. Stokes, in all their combinations and relations. The perfectly clear sound on percussion becomes a difficulty in our author's explanation of percussion; and he conjectures that either the diminution of air in the chest, by the swelling and effusion in the bronchi, does impair the sound, but in a degree imperceptible to our ears, or that there is a simultaneous dilatation of the air-cells, which may compensate for the air displaced by the state of the mucous tissue. But, if we suppose, according to another view before noticed, that the sound depends on the freedom of the parietes of the chest to vibrate, we can easily see why bronchitis, which does not alter the peripheral or external parts of the lung which are near the parietes, does not materially modify their vibrations. The following passage is a good specimen of the clinical application of principles of diagnosis, which the student will find admirably inculcated throughout this work.

"Yet, though percussion gives no direct result in bronchitis, its employment is of importance in the particular diagnosis. Thus, suppose that, after the existence of three or four days of fever, cough, hurried and difficult breathing, the chest still sounds well, the great probability is that the disease is bronchitis. The patient has had an acute inflammatory affection of the lung, and of but a few days' standing: this must be either bronchitis, disease of the serous membrane or of the parenchymatous tissue itself. Here the absence of dulness is of the greatest importance; for, were it a case of pleuritic effusion or of disease of the substance of the lung, the great probability is, that by this time a degree of dulness would be manifested: in the one case, the lung would be compressed, and its place occupied by a liquid effusion; in another, more or less obliteration of the air-cells would take place, from congestion or from inflammation. The absence, then, of dulness, with the existence of acute irritation of the lung, which has continued for several days, forms an important argument that the case is one of uncomplicated bronchitis." (P. 69.)

Bronchitis modifies the sound of respiration by, 1, the turgescence of the mucous membrane, a cause which principally affects the phenomena of the smaller tubes and air-cells; 2, the existence of an anormal secretion into the cavity of the tube itself; and, 3, the existence of spasm, the amount of which is exceedingly variable in different individuals. All these unite in forming the numerous varieties and combinations of Laennec's *sonorous*, *sibilous*, and *mucous râles*. The complete suspension of the sound of respiration in a part of the lung is another sign in bronchitis. Laennec ascribed this to an obstruction by mucus; our author thinks that spasm may have some effect in producing it. We do not see why any of the causes of the dry rhonchi, which are partial obstructions, when existing in a greater degree, may not become complete obstructions. Swelling of the membrane may do this, as we know it obstructs the nasal passage in recent coryza; concrete mucus may obviously do so likewise; and spasm may assist in producing the same thing, but, unless in the smaller tubes, we do not think it possible that any contraction of the circular fibres could close the bronchi. But these causes more frequently co-operate, both in the partial and in the total obstructions, and it would be refining too far to attempt to distinguish between them. So also with regard to the catarrhal rhonchi, we think that authors, Dr. Latham in particular, have attempted to make distinctions that are not found in nature. Thus, the sibilant rhonchus, so far from being, as the latter writer asserts, confined to the smaller bronchi and the more severe forms of disease, may be produced, by the above causes, in bronchi of any caliber, and when the affection is but slight and transitory. The universality of the rhonchi is a better index of the severity of the affection, yet Andral, with reason, objects to Laennec's assertion that the disease is almost always mortal when they are heard in every part of both lungs. (*Notes*, p. 49.) In the bronchial influenza of last winter, it was not uncommon to find the various rhonchi in every part of the lung: had this sign continued long, it probably might have confirmed Laennec's statement, as it did in a few cases of weak and broken-down constitutions; but it was generally transient, coming on at intervals, and leaving signs of only a more partial affection.

Dr. Stokes's observations on acute *secondary* bronchitis, accompanying continued fevers, are highly valuable, as they confirm and enlarge the views of Laennec on this subject.

"The occurrence of bronchitis in cases of typhus is not constant; and, even when it exists, it is often slight and easily manageable. But, on the other hand, the pulmonary system may be severely attacked, and death induced by asphyxia from excessive secretion of the bronchial membrane. We commonly meet with this severe form under two circumstances; the one where the symptoms are manifest and distressing, the other in which the disease is latent and insidious. But in one respect both these forms agree, namely, that, at an earlier period than in the idiopathic catarrh, secretion generally comes on in enormous abundance, and is too often the immediate cause of death. As far as I have seen, the great majority of patients in fever, who have died with what is called effusion into the chest, owe their death to this disease, which has been overlooked or insufficiently treated. This fact illustrates the want of proportion which commonly exists in typhous fever between the functional alteration and the organic change. With symptoms of an apparently trifling character we may, after death, find universal bronchitis, great congestion, or pneumonia.

"In many cases, as Laennec has observed, a bronchitis shall exist through the whole course of a fever, yet so slight as to merit little notice. But in all these cases we must pay a careful attention to the chest; for we know not the moment at which this trivial disease may assume a dangerous character: and hence, when we discover any increase in the bronchitic symptoms, we should immediately direct our attention to the lung, and, if possible, arrest the progress of the local disease." . . . . .

"This form of disease is commonly coexistent with more or less of gastro-enteric inflammation, thus forming one of the most fatal varieties of fever in this country. In some instances the disease predominates in the respiratory, in others in the digestive system; and I have often observed a remarkable alternation of this predominance of disease between the thoracic and abdominal cavities. Thus, suppose today we observe the breathing hurried and laborious, the cough troublesome, the expectoration difficult, and the stethoscopic signs well marked, the chances are that the abdominal symptoms are less severe, the abdomen is less swelled and painful, diarrhœa has ceased, the tongue has improved, and that characteristic prostration which attends gastro-enteric inflammation has remarkably disappeared. In two or three days, however, the abdominal symptoms return, with decided diminution of those of the chest, and in the course of a single case several alternations of this kind may occur. In such instances death generally takes place by asphyxia, and I have known cases in which the gastro-intestinal mucous membrane was found in so favorable a state as to leave little doubt that, as far as its organic change was concerned, the patient would have recovered but for the bronchitis. I think we may state, with respect to the pathology of the mucous membranes in fever, that, although the gastro-intestinal mucous surface may be, and often is, affected, while but little, if any, disease exists in the respiratory organs; yet that the converse of this proposition is seldom true,—a point of the utmost importance in practical medicine, as bearing on the general, local, and specific treatment." (P. 81.)

The physical signs of this secondary bronchial affection are those of ordinary bronchitis, but they are often obscured and complicated by signs of a congested state of the lung. Thus, there may be some dulness on percussion, particularly in the most dependent parts, and the rhonchi, from the same cause, may be very small and indistinct in these parts. The signs, in short, approach to those of pneumonia, and so does the pathological condition of the lung, which after death is found completely engorged and bordering on hepatization. M. Andral adds his testimony as to the frequent occurrence of latent bronchial affections during fevers.

"One of the most unexpected results of auscultation is the knowledge of the existence of this remarkable engorgement of the bronchial mucous membrane in almost every case of *dothinentery*, without at the same time cough, oppression, pain in the chest, or other symptom, enough to cause the suspicion of disease of the respiratory organs. A sibilant rhonchus, variable in intensity and extent, is the most common sign of this affection. Sometimes this is replaced by a deep sonorous rhonchus, and it is sometimes mixed with a mucous. But in the last case there is generally cough, and, as this symptom manifests itself, the rhonchus becomes more and more humid. Lastly, in certain cases we find, towards the posterior parts of the thorax, on one or both sides, a subcrepitant rhonchus, unaccompanied by any other manifest sign of pulmonary or bronchial inflammation. I have seen this last rhonchus continue many days in patients under *dothinentery*, who scarcely coughed once or twice in twenty-four hours, and whose respiration seemed quite unembarrassed." (*Notes, &c.* p. 71.)

Secondary bronchitis is of frequent occurrence also in the exanthematous fevers, and may constitute their chief danger. We owe to Broussais much of the knowledge of the important share which disease of the mucous membranes has in these affections; but the subject has been also well investigated by Billard, Guersent, &c. on the continent, and the occurrence of a formidable kind of bronchitis in measles has been long since noticed by Dr. Hastings and others in this country. The bronchitis of measles and scarlatina has generally a more sthenic character than that of typhus; and it is more common, in the former diseases, to find the serous membranes involved.

Dr. Stokes agrees with Dr. Graves in thinking that the study of chronic bronchitis, which is secondary to other chronic diseases, has been too much neglected. Thus, as bronchitis accompanies those general affections of the system called fevers, so a similar condition of a chronic kind may occur in the gouty, scrofulous, syphilitic, and scorbutic disorders. We are not, however, prepared to admit an entire analogy between these two sets of cases. That fevers, which essentially involve the disturbance of a great many functions, especially those of circulation and secretion, should produce local congestions, inflammations, and disordered secretions of the great mucous surfaces, is as consistent with theory as it is proved in experience: but that diseases, which necessarily produce no such constitutional disturbance, and manifest their constitutional character chiefly by developing local disease in distant parts, or by modifying diseases that are otherwise produced,—that such diseases should severally, of themselves, excite similar congestions or inflammations in the mucous membranes, is as questionable in theory as it is unconfirmed by experience. When gout is attended with fever, this fever may develope a bronchitis, and the prevailing diathesis may give a character to it; or, when a scorbutic person takes cold, the bronchitis that succeeds may derive a character from the condition of the body; but it is not the gouty or scorbutic contamination in these instances that excites the bronchitis; and, although we may have gouty, syphilitic, and scorbutic varieties of bronchitis, we cannot think that these are generally associated with those respective disorders, as bronchitis and pulmonary congestions are with fevers. It is, however, highly important to mark these varieties, and to be aware of the aggravations which they produce. Dr. Stokes dwells chiefly on the bronchitis which sometimes accompanies syphilitic affections. This is sometimes acute, developed by a fever, and subsequently removed or lessened on the appearance of a copper-coloured eruption on the skin. The chronic form shows itself in association with periostitis, sore throat, debility, night sweats, and other evidences of a state of syphilitic cachexia, in which the occurrence of cough may cause a suspicion of tubercles. If, however, on careful examination, no sign of these be found, mercury will generally soon shew its beneficial effects.

Dr. Stokes concludes the history of bronchitis with some very useful remarks on the mode of distinguishing the sympathetic cough, or that depending on irritation of distant organs, especially the gastro-enteric membrane. In such cases, the diagnosis will chiefly depend on the absence of signs of pectoral disease or any more direct source of irritation, as in the larynx, fauces, &c., and the existence of symptoms of an offending cause elsewhere: this is not unfrequently worms in the intestines. An instance is given where a severe form of intermitting bronchitis, which entirely resisted the ordinary treatment for many days, was cured by turpentine, syrup of cowhage and castor-oil, which brought away large quantities of thread-worms.

*Treatment of Bronchitis.* In the bronchitis of dentition, Dr. Stokes recommends free incision of the gums, mild purgatives, and ipecacuanha in minute doses. He ascribes many cases of the severe form of infantile bronchitis to the habit of keeping children within doors for months from their birth, which renders them very susceptible of bronchial irritation. "The necessity and safety of bringing the young infant into the open air in the course of a few days after its birth, is not sufficiently known, and many lives are thus sacrificed to an absurd, ignorant, and destructive prejudice." This may be in some measure true, but we think that it should be qualified with an exception in case of the winter season and cold weather. We are quite sure that many lives are lost in the injudicious attempts of some to render infants hardy before they possess that power of resistance to cold on which hardiness depends. Dr. Stokes must be well aware of the experiments of Milne Edwards on the power of animals to produce heat according to their age, and also of the statistical results of the early registration of infants in France, in relation to this point.

For severe cases of puerile bronchitis, Dr. Stokes chiefly recommends tartrate of antimony, and, if the patient be robust, bloodletting, especially by leeches. To stop the bleeding from leech-bites, he recommends a fine pencil of nitrate of silver to be inserted lightly but rapidly to the bottom of the puncture, the blood having been first wiped out with lint. In the bronchitis of adults, also, he trusts much more to local than to general bloodletting, and thinks it more effectual when made in the upper than in the lower parts of the chest, as originally recommended by Broussais.

> "It is scarcely necessary to remark, that, under certain circumstances, local bleeding may be repeated, even in an advanced stage of the disease. As, for instance, suppression of expectoration, when this coincides with increase of fever and irritation; increase of dyspnœa, when this is not produced by over-secretion, a point easily determined by the stethoscope; and, lastly, the occurrence of local dulness, which, in cases of intense bronchitis, may occur, and is owing to congestion of the substance of the lung." (P. 110.)

M. Andral gives a valuable note on the employment of bloodletting in bronchitis. Without adopting the statement of Laennec, that he had never found bleeding indicated in the suffocative form of catarrh, he thinks it very questionable that this measure can in many cases exert any control over the engorged state of the bronchi, or the profuse secretion which they contain. He has seen death certainly hastened by this measure, and he concludes with a remark which his Broussaian countrymen would do well to attend to:

"It is not only because a part is red, swollen, or its secretions changed, that bleeding is indicated; for this anatomical condition is not always the result of the same lesion; and there is a case of this sort which is better removed by bark than by blood-letting." (*Notes*, p. 75.)

The remedy next in importance, in Dr. Stokes's estimation, is tartar emetic; but, in its exhibition, "certain considerations must always be attended to:" and, generally, the more sthenic the disease, and the greater the exemption from complications, especially with abdominal disease, the more safe and efficacious will be the remedy.

"It would seem, however, that mere prostration should not necessarily prevent us from having recourse to the remedy. Indeed, cases are recorded in which the patient, at the time he was ordered the tartar emetic, was almost in *articulo mortis*. I have never seen such a case, but have often found, in the advanced stages of acute diseases of the pulmonary parenchyma and mucous membrane, when other means have either failed or proved in a great measure inefficient, and where the patient was necessarily much debilitated, that the exhibition of the tartar emetic was followed by the happiest results. My experience at present leads me to conclude that, where the debility is merely traceable either to the disease or to antiphlogistic treatment, and not the result of its complication with decided abdominal inflammation, we may often have recourse to the antimonial solution; and we shall find that, when managed with judgment and caution, it will then, perhaps more than at another time, exhibit its almost specific power on the capillaries of the lung." (P. 112.)

The mode of exhibition adopted by Dr. Stokes at the Meath Hospital does not much differ from that of Laennec; viz. Tartr. Antimonii, gr. vj.; Aq. Cinnamomi, ℥vj.; Tinct. Opii acetatis, gtt. xij. Half an ounce of this is given every hour or second hour, so that the whole six grains may be taken in twenty-four hours. Three or four grains only will, however, often produce marked benefit. The medicine is found to act much in the manner described by Laennec, but Dr. S. has seldom found it necessary to increase the quantity beyond eight or ten grains in the twenty-four hours; but in all cases it must be left off gradually, otherwise the symptoms are apt to return. Our own experience agrees with these statements, and still more fully with those which follow.

"As far as I have seen, the effect of this medicine on bronchitis is twofold. It may either, as it were, cut short the inflammation, so as to leave hardly a symptom or sign behind it; or it may cause its early passage into the second or secretive stage. In the first case, the oppression and wheezing cease, the cough becomes trifling, the lividity disappears, the pulse falls to its natural standard, and the respiration is found everywhere pure, equal, and healthy, with the exception perhaps of a slight sonoro-mucous rale, which is now and then audible; the patient recovers his appearance, and declares that he is quite well. In the second case, after the use of the remedy for several days, we find the patient looking pale and miserable; he perspires copiously, and has often a rapid small pulse; the breathing, though less difficult, is hurried; and the cough, though less painful, is so frequent as to allow of but little rest. It is followed by a copious expectoration of opaque mucus or of a muco-purulent secretion. On percussion, the chest sounds clear, but the respiration is generally marked by mucous rales, of various intensities, in some cases combined with the sonorous, in others passing almost into the crepitating character. At this period antiphlogosis can be used no longer, and a cautious but decided employment of the stimulating and tonic treatment must be had recourse to. But, even in this instance, though the exhibition of the tartar emetic has not, as in the former case, restored the lung to a state of health, yet it has not been without its advantages; inasmuch as experience shows that now the exhibition of stimulants and tonics will have the best possible effect. This fact, among many others, seems to me illustrative of a general rule in therapeutics, that in almost all local diseases the successful employment of stimulation depends on the previous use of a general or local antiphlogistic treatment." (P. 113.)

The art of discerning the transitions of disease from sthenic to asthenic, and of adapting new kinds of treatment to these changes, belongs exclusively to the skilful practical physician. No study of morbid anatomy can teach it; for morbid anatomy tells nothing of those conditions of the whole body, on the knowledge of which this art chiefly depends. Here, too, the mere stethoscopist will be at fault; for, although the physical signs may still inform us accurately of the mechanical condition of the injured parts, they will not tell us whether the vital action with which that condition is connected is of a sthenic or of an asthenic kind, nor what is the state of the general strength in relation to therapeutic agents. There is still much obscurity about the signs and symptoms which guide the experienced physician in these cases: he may acquire the art of deciding successfully, without being able to describe distinctly the circumstances that guide him in his decisions. In this respect the art is, to a certain extent, incommunicable, and can be derived only from long experience. But, probably, as our knowledge of the body and of its healthy and diseased properties increases, even these illusive and indefinite signs will become more intelligible and distinct, and, being accurately analyzed and explained, they may be made to furnish in clinical instruction that knowledge which can now be only obtained by long and painful experience. In his reiterated observations on the necessity of a change of treatment in the course of bronchitis, and on the circumstances in which a stimulating plan becomes indicated, we find Dr. Stokes endeavouring to give greater precision to this difficult part of practical medicine; and his remarks are well worthy of the attention of the young practitioner. The following propositions sum up those points which relate most directly to the treatment of pulmonary disease:

"First. That in some cases an antiphlogistic treatment may cut short the disease in its first stage; but that, in most instances, particularly in the affections of mucous membranes, its effect is to bring on the occurrence of the second stage.

"Second. That the principal circumstance on which the success of stimulant depends, is their having been preceded by antiphlogistic treatment.

"Third. That in many cases disease will continue for a great length of time, and yet (as shown by the result of treatment,) be in its first stage. Although chronic as to its period of duration, it is still acute when tested by the effect of treatment.

"Fourth. That this result is most frequently seen under the following circumstances: (*a.*) Cases of local disease, with but little injury to the general health. (*b.*) Diseases of tissues, where there is but little relief by secretion. (*c.*) Diseases of organs which have been neglected, or exasperated by too early stimulation.

"Fifth. That, in many cases where the disease has been neglected or exasperated, it will be necessary to precede all stimulants by an antiphlogistic treatment, either general or local." (P. 117.)

Of the local or specific stimulants, Dr. Stokes thinks that the decoction of senega, with carbonate of ammonia, camphorated tincture of opium, and tincture of squill, is the most effectual. "Under its influence, the expectoration diminishes without increase of dyspnœa; the pulse becomes slower and fuller; the respiration in the upper portions of the lung becomes pure; and this change, extending from above downwards, we may find that, in a very few days indeed, all morbid signs will disappear from the lung." The success of this, and especially of more stimulating remedies of this class, will depend on their being employed at the proper time, when the inflammatory stage of the disease has in great measure subsided. It is a useful measure to conjoin with them external counter-irritants, of which blisters are the chief. Dr. Stokes recommends in the apyrexial forms of bronchitis, as well as in phthisis, sponging the chest with a liniment composed of Sp. Terebinth. ℥iij.; Acid. Acetici, ℥ss.; Vitell. Ovi. j.; Aq. Rosar. ℥ijss.; Ol. Limon. ʒj. This, he thinks, acts not only as a counter-irritant, but also by being absorbed, and stimulating the mucous membrane.

Among the stimulants which are useful in the apyrexial and chronic bronchitis, the terebinthinate medicines and balsams are named, and Dr. Stokes suggests that strychnia, which is so efficacious in analogous affections of the gastric mucous membrane, might be beneficial here, not only by modifying the secretion, but in stimulating the bronchial muscles to contract. Quinine and iron may be often advantageously employed where the powers of life are low, and where the antiphlogistic plan has been pursued.

In the secondary bronchitis of typhus, the antiphlogistic means must not be employed so boldly or so long, whilst stimulants and blisters may be soon and freely resorted to; and no such complete or permanent effect is to be expected from the use of any measures, as in the idiopathic disease. We regret that we have not space for inserting more extracts from this part of the section, which, we think, comprises a much better account of the diagnosis and treatment of bronchitis than any previously published.

The organic changes of the tubes and air-cells, considered in relation to bronchitis, are, 1, narrowing of the caliber and obliteration; 2, dilatation; 3, ulcerative destruction of the tubes; 4, enlargement of the air-cells; 5, atrophy of the lung. Dr. Stokes does not contend for the inflammatory origin of these in every case, but the bulk of evidence is in favour of the connexion of these lesions with an inflammatory process in a majority of cases. We believe so, too; but, until the connexion be more fully traced and acknowledged, we question the expediency of classing all these cases together.

Adopting Reisseissen's account of the structure of the lung, which has been confirmed by M. Reynaud, both M. Andral and Dr. Stokes make the remark, that the bronchial tubes progressively subdivide, but never anastomose; their ultimate ramifications terminating in *culs de sac*, which are the air-cells; and on this character of structure several of their pathological changes depend.

Obstruction and obliteration of the bronchial tubes have been mentioned by writers on morbid anatomy, but M. Reynaud first described them as a not uncommon pathological condition; without, however, speaking positively as to their causes or symptoms, Dr. Stokes thus takes up the subject:—

"It is obvious that, when inflammatory action seizes on a bronchial tube, its effect, considered anatomically, will vary according to the diameter of the canal. In the larger tubes, whose parietes are guarded with strong cartilaginous plates, nothing but a great local hypertrophy of the mucous membrane could cause an obliteration; while, in the minuter tubes, whose perviousness is not so provided for, the same process would much sooner produce obliteration." . . "In the larger tubes we find a vascular mucous membrane endowed with villosities and glands; but, as we advance into the substance of the lung, this tissue gradually loses its original characters, until, at its ultimate point, if it be not completely serous membrane, it closely approaches to it in appearance and function. If we now add these considerations to the preceding, we get at once a sufficient explanation of the point in question. As M. Reynaud remarks, we may expect the plastic inflammation the more the affected tissue approaches to white structure; and hence another cause of the greater liability of the minute tubes to obliteration." (P. 137.)

"It would appear that we may consider this obliteration of the bronchial tubes in two points of view: first, as commencing in the finer, and proceeding by continuity of disease to the larger tubes; and, secondly, as the result of obstruction of a large trunk, and the consequent obliteration of the tubes to which it gave birth, by a process similar to that observed in arteries after ligature. Of these species, the first is the most frequent and important; and I cannot help thinking that its investigation will not only go far to clear up the long-controverted point as to the nature and origin of tubercles, but also throw light on other subjects of thoracic pathology." (P. 141.)

Dr. Stokes objects to M. Reynaud's distinction between the cases in which obliteration is caused by simple adhesion of the sides of the tubes, and those resulting from obstructing matter effused within them. But we are inclined to think that the distinction is a just one, inasmuch as the former is secondary to some other lesion: while the latter is more simply the result of a plastic inflammation of the bronchial tubes. And here we would also remark, that neither of these authors have sufficiently adverted to another cause of obliteration of both tubes and cells, which our examinations lead us to believe to be far from uncommon. Dr. Stokes has fully dwelt on obliteration of some tubes as a cause of the dilatation of those tubes and cells adjoining them, but he has not noticed the converse of this, the obstruction and ultimate obliteration of tubes and cells by the pressure on them of other tubes and cells which have undergone dilatation. Yet not only this must tend to such encroachment on the healthy tissue, but every other lesion that can permanently change the relative position of the parietes of the tubes and vesicles with regard to each other. Thus, Andral observes, that tuberculous, and we would add other deposits, partial indurations, interlobular thickenings, and other similar lesions, must more or less press on the adjoining tissue; and, in so doing, they often obstruct some of the bronchial tubes which pass through it, which may become obliterated in consequence. The cells to which these tubes lead, no longer receiving their supply of air, become collapsed also; and, to fill their space, the adjoining tubes and cells must be unnaturally distended: here we see an additional reason for the common complication of tubercular deposits with pulmonary emphysema and dilated tubes.

Dr. Stokes introduces the subject of *dilatation* of the bronchial tubes by describing the analogy between these tubes and the arteries, and deducing the existence of dilatation of the former from the supposed parallel case of aneurism. Now, we think that this supposed analogy is not only imperfect, but also calculated to mislead the mind as to the true character of dilatation of the bronchi. There is such a difference between the general and special anatomy and physiology of the arteries and those of the bronchial tree, that the assistance derived from analogies of their respective pathological states must depend rather on their mechanical resemblances as tubes, than on any close similarity of structure. With regard to the cause of dilatation of the bronchi, Dr. Stokes supposes, with Williams and Roche, that inflammation, which by diminishing the cohesion of elasticity of their tissues, makes them yield to the distending air in cough and respiration, is the chief; but he adds that this inflammation has another effect, hitherto unnoticed, which may lead to the same result; it paralyzes the bronchial muscles.

"There can be no doubt of the fact, no matter how we explain it, that, where muscular structures are in close connexion with other tissues which are inflamed, their functions suffer, and we observe, first, an increase of innervation, as shewn by pains and spasms, and next, a paralysis more or less complete. When we come to speak of empyema, diaphragmitis, and inflammation of the heart, we shall see of what importance these considerations are. At present, it appears that we may hope to elucidate some points in the symptoms and treatment of bronchitis by having recourse to this view. May not this paralysis explain the difficulty of expectoration in certain cases; the stasis of matters in the tubes, and the liability to asphyxia in bad catarrhal fevers? and we might further enquire how far its existence should lead us to modify our treatment, and seek for some agent which would stimulate the bronchial muscles to contract. Abercrombie relates a case of distention of the bowels in which galvanism had the best effect, and I have already alluded to the use of the same agent in pulmonary disease by Drs. Philip and Forbes. Now, as the lung derives a large portion of its nervous supply from the cerebro-spinal system, we might hope, by the exhibition of such remedies as strychnine, to act beneficially upon it when its innervation was injured." (P. 151.)

These views, although at present theoretical, are quite rational and highly deserving of the attention of the scientific practitioner. We have seen many facts confirmatory of the position first maintained by Dr. Abercrombie, that inflammation frequently causes in adjoining muscular structure, first, increased and afterwards diminished action; and the diminution may in some cases amount to a perfect and permanent paralysis. We would, however, also remark, that there may be diminished action, and even paralysis without previous inflammation; and, with regard to the particular case under consideration, we have seen dilated bronchi with an uncommon development of the circular fibres.

The remainder of this portion of the section is devoted to the complications and diagnosis of dilatation of the bronchi; it contains some good matter, but lacks arrangement and condensation. We can only give the recapitulation, which our readers will perceive is not a brief one.

"1st. That the cases of this disease which have been described by authors may be divided into three classes: (*a*) cases in which symptoms of chronic catarrh, with copious expectoration, have existed for a number of years, varying from ten to fifty or even more, and without the constitutional symptoms of phthisis. (*b*.) Cases presenting the symptoms of phthisis, in which the constitution suffers severely; the disease may last from five months to five or even ten years. This last case has been principally observed in adults. (*c*.) Cases which may be termed acute. These are to be observed in children after hooping-cough, and the disease has occurred in the space of three months.

"2d. That we meet this affection as an uncomplicated disease, or in conjunction with other lesions, of which obliteration of the bronchi and tubercle are the most common.

"3d. That dilatation of the bronchial tubes may be accompanied by an atrophy of the air-cells, and thus the affected side of the chest be diminished in volume.

"4th. That, in the same case, we may observe a predominance of dilatation in the bronchial tubes of one lung, and of the air-cells in the other.

"5th. That the continuous dilatation may affect a single tube, without presenting any marked physical signs.

"6th. That we may even have numerous small dilatations without other phenomena than those of ordinary bronchitis.

"7th. That, when the continuous dilatation is decided and extensive, the phenomena which have been observed are the flowing respiration and extended resonance of the voice. In some cases, too, the veiled puff has been observed by Laennec.

"8th. That when the local dilatations are decided, the phenomena are those of suppurating cavities communicating with the tubes.

"9th. That, although it is extremely difficult, on account of the similarity of the physical signs, and in some cases of symptoms, to distinguish this disease from phthisis with suppurating cavities, yet, by observing the mode of combination, and the succession of the signs, the rate of increase of the cavities, and the connexion of these with the history of the case, we may, in some cases at least, arrive at a diagnosis which shall be correct.

"10th. That, where a number of tubes are detailed in one lobe, the case may be distinguished from tuberculous anfractuosities by the clearness of sound on percussion.

"11th. That, in cases where we have had an opportunity of examining the patient from an early period, the fact of dulness not having preceded the signs of a cavity may enable us to distinguish the disease from phthisis.

"12th. That, in the same manner, the combination of extensive tracheal respiration with clearness of sound, seems to be diagnostic of dilated tubes." (P. 170.)

Dr. Stokes justly objects to the term *emphysema* of the lungs, applied by Laennec to general dilatation of the air-cells; for emphysema being generally understood to signify effusion of air, is not applicable to many cases of the affection in question in which there is no evidence of any entry of air into the interstitial tissues; and the essential character of which is an enlargement of the natural vesicles of the lungs. But when he says, "the lung becomes enlarged," he excludes one of the forms of the disease, much dwelt on by Andral, that resulting from atrophy or wasting away of the solid parietes of many of the cells; so that, without dilatation or enlargement of the whole lung, many cells break down into a few large ones, and present that coarseness and lightness of texture which is remarkable in the lungs of old people. Dr. Stokes admits that the disease may originate, as supposed by Laennec and others, in the obstructions to the exit of air from the cells distended in forcible inspirations, occasioned by the swelling of the tubes or by viscid mucus in them; but he thinks that spasm and irregular action of the circular fibres may be an additional cause of obstruction, tending to preserve a dilated state of the cells. Dr. Williams has suggested another probable cause in the moveable pellets of viscid mucus plugging up certain tubes at the beginning of inspiration, so that all the air introduced by this act must pass into the adjoining tubes which remain open, and over-distend the cells to which they lead. We think that this latter explanation may be extended further to the still commoner case of partial obstruction of the tubes: for, if the air at each inspiration finds much readier access to some cells than to others, the distending force of each inspiration (particularly of those that are sudden,) will be more felt by these cells, which will consequently become more distended. To render this distention permanent from whatever cause it may proceed, there must be such a continued application of the cause as to make the natural properties of the tissue adapt themselves to it, or some morbid modifications of these properties; such as loss of elasticity, rupture, thickening, &c., which may at once fix the cells in their distended state. Further, there is a remark of Laennec, which is worthy of attention, as it seems to us to suggest another cause of dilatation of the air-cells. In the case of persons suffocated by the gases of cess-pools, he has remarked the lungs to be very large, although perfectly crepitous, and asks whether this may be from a general dilatation of the air-cells, a primary form of emphysema. Mr. Swan, in his experiments on the nerves, describes an emphysematous condition of the lungs to ensue after the eighth pair of nerves in the neck have been divided. Do not these facts, to which we could add others, render it probable that the sensation and motion of the bronchi are essential to the perfect performance of the expiratory act, and that a paralysis of these properties, by rendering this act imperfect, may be a cause of dilatation of the air-cells?

We can only give, in this place, Dr. Stokes's general conclusions respecting emphysema; they are as follows:

"1st. That the disease consists essentially in an enlargement of the air-cells. 2. That the rupture and coalescence of several cells is not a constant occurrence. 3. That the disease increases the volume and rarefaction of the lung. 4. That it may occur uncomplicated with any affection except bronchitis, or exist along with other diseases which are equally chronic. 5. That it may coexist with great dilatation of the tubes. 6. That it may be partial or general. 7. That percussion gives a morbidly clear sound when the disease has attained a certain extent. 8. But that the cells may be so enlarged as to give feebleness of respiration without change on percussion. 9. That the physical signs of bronchitis which occur, though pointing out the existence of disease in the smaller ramifications, are not characteristic of the affection. 10. That the stethoscopic indication is the want of proportion between the sound of vesicular expansion, the results of percussion and the efforts of inspiration. 11. That a most important source of physical signs is to be found in the increased volume of the lung. 12. That this increase of volume can be ascertained by measurement of the chest, by the displacement of the mediastinum, by the depression of the diaphragm, and by the lateral displacement and the depression of the heart. 13. That although in this disease, as in empyema, there is pressure from within, yet that it differs from the latter affection in the absence of paralysis of the inspiratory muscles, as shewn in the comparative states of the intercostal muscles and diaphragm. 14. That the physical signs from auscultation are greatly modified by the degree of yielding of the thoracic parietes, the characteristic feebleness of respiration appearing to be directly as the amount of resistance to the increased volume of the lung. 15. That, in the same way, the signs resulting from the displacement of the mediastinum, heart, and diaphragm, will vary with the amount of resistance of the thoracic parietes, and be more obvious the greater the resistance. 16. That the intercostal spaces are not protruded in this disease, but preserve their relative positions with respect to the ribs. 17. That the cases of the disease may be divided into two classes, viz. those in which the diaphragm is unaffected, and those in which it is depressed. 18. That, in the first class, the abdomen is collapsed, and without tumefaction or dulness of sound in the epigastric or right hypochondriac regions. In these cases, the heart is found in its natural position. 19. That, in the second class, the reverse occurs: the liver is depressed and the heart so displaced, as that it has been found to pulsate so low as the ninth intercostal space. The postero-inferior portions of the chest sound clear even to the last rib. 20. That, under these circumstances, the diaphragm being flattened, its contraction acts in diminishing the circumference of the trunk in the region between the eighth and tenth rib, so that we observe expansion of the upper portion of the chest and of the umbilical region, while the portion above mentioned manifestly contracts. 21. That the volume of the lung varies remarkably at different periods. 22. That, when it is greatest, all the physical signs are most evident. 23. That the cause of its increase is an exacerbation of the bronchitis. 24. That, under treatment calculated to remove bronchial irritation, the vesicular murmur may return, and the volume of the lung is diminished. 25. That these facts are in favour of the opinion that the disease is susceptible, if not of cure, at least of great alleviation." (P 201.)

The researches of Dr. Stokes on the pathology of pulmonary emphysema are more satisfactory than those of M. Louis and the late Dr. Jackson. We shall give an analysis of M. Louis's paper in our notice of the work in which it appears, (*Mem. de la Soc. Med. d'Observation*, t. 1.) and will merely state that he doubts the connexion of emphysema with pulmonary catarrh, because the explanations of Laennec were found inapplicable to the cases which he examined; no mucus being ever found in the dilated air-cells, and the dilatations affecting especially the margins of the lobes, which are rarely, if ever, the seat of pulmonary catarrh. But the views which we have noticed extend beyond those of Laennec; and it may be remarked, that the marginal or terminal cells of the pulmonary tissue will be especially liable to distention from those violent but partial inspirations which have been supposed to occasion pulmonary emphysema. The disease of the heart and signs of obstructed circulation, noticed by Louis, as well as by preceding writers, as frequent concomitants of emphysema, we consider to be in a great measure caused by the mechanical effect on the great vessels, of the rigid and distended lung which is continually pressed on them by the *forced expirations*, so characteristic of the advanced stages of this disease. Laennec had a more extended knowledge of the pathology of pulmonary emphysema than M. Andral, and subsequent writers seem disposed to allow him; for he distinctly describes loss of elasticity, hypertrophy, and occasional atrophy and rupture of the cells, as connected with the permanent forms of the lesion. We do not find in the "notes" any important addition to the original matter of Laennec, except the remark from M. Louis regarding the state of the subclavicular region in emphysema of the lungs. This presents a prominence which contrasts remarkably with the hollow which is usually seen above and below the clavicles. M. Louis considers this state peculiar to those who have emphysematous lungs; and in this he is confirmed by M. Andral.

We can only notice very briefly the subject of *Pneumonia*. Dr. Stokes thinks that there is no absolute line of distinction between bronchitis and pneumonia, and that it would hardly be a misnomer to call pneumonia a bronchitis of the terminal tubes. This is too wide a question to enter on here, but we may express a doubt whether such an approximation of two diseases that are in most instances distinct in character and relation to remedies as well as in seat, be expedient or philosophical. It is quite true that a great portion of the parenchyma, which is the seat of pneumonia, consists of the minute bronchi and cells; but, besides the difference which the intervesicular texture and plexus of vessels produce, the circumstance of the inflammation pervading tissues closely packed together instead of affecting a membrane lining a number of separate tubes, is enough to constitute a generic difference which as it affects the characters, so it should be represented in the names of the two diseases. It should never be forgotten too that the function of the parts affected in bronchitis is quite distinct from that of those affected in pneumonia: the bronchial tubes merely convey the air, but their finer ramifications and terminations are the seat of the changes which this air effects in the blood: the former are supplied merely by their nutrient vessels, the bronchial arteries; the latter have besides the great plexus of the pulmonary vessels distributed upon them through which the whole blood of the body passes. These points are strongly dwelt on by the writer of the article "Pneumonia" in the Cyclopædia of Practical Medicine, who considers the essential seat of pneumonia to be this great pulmonary plexus, but that its anatomical and perhaps its pathological characters may vary according as these inflamed vessels extend their effects chiefly to the air-cells or to the intervesicular texture. The late researches of MM. Hourmann and Dechambre on the pneumonia of old people at the Salpetrière, and which we have noticed in our preceding numbers, seem to favour this view. They have shewn that there is a non-granular form of hepatization, in which the intervesicular and interlobular tissues are the chief seats of effusion, and this is just what Dr. Williams, the author of the article just noticed, has described.

Dr. Stokes thinks that there is a stage of pneumonia prior to that of engorgement, described as the first by Laennec, in which the pulmonary tissue is drier than usual, not at all engorged and of a bright vermilion colour. We believe that we can confirm our author's opinion on this subject, having repeatedly found, in the vicinity of a tissue in the engorged degree of inflammation, a florid vascularity without any liquid effusion; and in two or three cases we have observed with the general symptoms of an attack of pneumonia, a louder or puerile respiration, which is supposed by Dr. Stokes to accompany this degree of inflammation, which preceded the occurrence of crepitation. Such a sign is what might be expected: the enlarging vessels diminish the caliber of the minute tubes and cells, and bring them into the condition of the tissue of a child's lung: a farther degree of diminution from the same cause and from the addition of the viscid mucous secretion constitutes the elements of the crepitation of the stage of engorgement. We suspect that this early sign of inflammation is of very short duration and is to be heard without crepitation only during the first two or three hours after the attack. In fact it may be regarded as a degree of the stage of engorgement rather than as a separate stage; for there is no evidence to prove that, in the stage of engorgement, blood is, as Dr. Stokes assumes, effused into the air-cells. It is quite important, however, to know of the existence of a first degree of inflammation without crepitation, although our observations are rarely early enough to discover it. A local puerility of respiration, accompanied by fever and the general symptoms of inflammation in the chest, may be therefore taken as the indication of an incipient pneumonia.

M. Andral, as well as Dr. Stokes, has met with cases presenting the rational symptoms of pneumonia without crepitation or any of the ordinary physical signs. The former views these as cases of central pneumonia, and that, although the sound of vesicular respiration is audible in every part of the chest, yet that it presents this character, that the expiration is unusually distinct, and continues to be so until resolution is completed. *Notes*, p. 156.

We have a word to offer on the following passage:

"On the subject of Laennec's first stage, it is to be observed that it does not necessarily precede hepatization. We may have complete solidity produced in a lung that has never presented the crepitating rale, and the disease passes on into the stages of suppuration and abscess. This circumstance, so important in diagnosis, is met with in certain cases of the typhoid pneumonia, in which a sudden and extensive congestion of blood affects the lung. It may then occur that a lobe which to-day was perfectly permeable, and presenting no morbid signs, shall, in twenty-four hours, be solidified, and present dulness, with absence of vesicular murmur, broncho-phonia, and bronchial respiration." (P. 311.)

We can testify to the truth of these last facts, but we interpret them differently. We would rather say that these cases were still in the first stage, and that the second stage, hepatization, does not necessarily precede the third, suppuration. That we may not be misunderstood in the terms used, we would restrict the word hepatization to mean that solid state of the lung which, in weight and firmness, really does resemble liver; and we cannot agree with Dr. Stokes that this solidification "arises, not from any deposition of lymph, but merely from an excessive congestion of blood;" and that, therefore, this is only the maximum of the stage of engorgement. No one can closely examine a truly hepatized portion of lung without perceiving in its lighter colour, especially in the granulations, the sign of a preponderance of solid albuminous matter, which is never to be found in the merely engorged lung, and which mere congestion of blood could not produce. But we do believe, from repeated observation, that mere congestion or engorgement of blood in the lung may, when in excessive degree, produce dulness on percussion and bronchial respiration, and we conclude that Dr. Stokes's examples of solidification were of this kind. We have found a condition presenting these signs, not only in fevers of the congestive and ataxic kind, but also in the advanced stages of diseases of the heart, come on in the course of a day, and in a few instances disappear as rapidly; but we cannot suppose with Dr. Stokes that no crepitation accompanied the commencement of this congestion; although its rapid increase produced in a few hours a total obstruction of those tubes which are the seat of this crepitation.

Dr. Stokes considers that the rarity of pneumonic abscess has been over-rated. He gives a very interesting example in which the patient recovered from the abscess, but dying of pneumonia a year after, a cicatrix was found in its place. He describes three forms of pneumonic abscess, which we may state thus: 1, the true phlegmonous encysted abscess; 2, purulent cavities communicating with the tubes without cyst; 3, intervesicular or interlobular suppuration, in which "the lung lies bathed in pus, and we have an abscess under the pleura, but external to the lung."

The author's observations on the symptoms and general diagnosis of pneumonia are excellent.

"The true source of diagnosis is our finding the combination of irritation of the respiratory system with the physical signs of pneumonia; of which signs it may be said, that although, taken singly, any of them may occur in other affections, yet that, in pneumonia, their mode of succession is quite characteristic." (P. 320.)

Dr. Stokes has not found the viscid rusty sputa at all constant in pneumonia.

"But, in the suppurative stages, the expectoration is generally characteristic: it then occurs under two forms; in the one we observe a purplish red muco-puriform fluid, while in the other we find that the matter coughed up has all the characters of the laudable pus of authors. It is of a light yellow colour, perfectly homogeneous, and of the consistence of cream. I have never seen this expectoration, unless in the suppurative pneumonia, and it forms almost the only instance in which the expectoration of pure pus is met with." (P. 321.)

We have seen pure liquid pus expectorated in considerable quantities in a case of uncomplicated bronchitis. Dr. Stokes very justly remarks, with regard to bronchial respiration as a sign of hepatization, that for its production there must be expansion of some part of the affected lung; otherwise, there would be no passage of air through the bronchi. This remark does not, however, extend to bronchophony, when that is to be heard.

Typhoid pneumonia is described by our author as of frequent occurrence in Dublin, being at times almost epidemic. That complicated with typhus fever is remarkable for its latency. There may be no general symptoms of its presence, and the aid of the stethoscope is necessary to detect the presence of the disease. But, equally as with typhus fever, gastro-enteric and erysipelatous fever, pneumonia of the same kind may occur with delirium tremens, from excess. This has been noticed, in the article in the Cyclopædia before quoted, as the direct effect of excessive indulgence in intoxicating liquors, especially when conjoined with exposure to cold.

Dr. Stokes's observations on the treatment of pneumonia are well worthy of attention, but we doubt much if some of them will be found generally applicable. The following remarks, for instance, are probably more true in relation to the poorly-fed peasantry of the sister kingdom, than with the labourers of this country.

"I find the bold and repeated use of the lancet to be unnecessary in the great majority of cases, and I am convinced that in general a single, or at most two bleedings, will be sufficient. Out of many hundred cases, I have had only one in which it was necessary to bleed more often than twice: in this instance there was complication with hypertrophy of the heart. The true principle seems to be, that general bleeding is to be considered only as a preparative for other treatment, and not the chief means of removing the disease." . . . "I am most anxious to press the importance of local bleeding in the treatment of pneumonia, for I consider it as the principal remedy. For this purpose either scarification or leeches may be employed; but, if the latter be used, I would in all cases recommend that the cupping-glasses should be employed in conjunction with them. In this way the general fever and arterial excitement having been previously reduced by the lancet, we may, directed by the stethoscope, continue, day after day, to detract blood from the affected part, while the patient's strength can be supported by food, and even wine, if necessary. In the treatment of the typhoid form, the best practice is to use wine in conjunction with local bleedings." (P. 343.)

We do not often find that, in sthenic pneumonia of more than twenty-four hours' standing, the general fever and arterial excitement can be reduced by a single bleeding; and, although we fully agree in the beneficial effects of local bloodletting, we have in most instances found venesection much more effectual during the increase and continuance of the crepitating rhonchus with general inflammatory fever. There is a suddenness of impression produced by general bloodletting through a large orifice, that, if judiciously applied in the early stage of pneumonia, tends to relieve the congested lungs far more effectually than any drainings from leech-bites. Extensive cupping may perhaps do nearly as well, but it is more painful and less manageable, and we think is better as an adjuvant in this stage. We agree with Dr. Stokes, that there are few cases in which we must not at once add to bloodletting the aid of either the antimonial or the mercurial treatment, and we believe the circumstances to determine our choice between these have been truly stated by him.

"That the success of the antimonial treatment depends on, or is favoured by, the inflammatory character of the fever, the early stage of the disease, the absence of complication with other diseases, the fact of the patient having borne bleeding well, and the firmness of the coagulum: the more the case presents these characters, the greater will be the likelihood of the tartar emetic acting favorably. But, in the typhoid, secondary, and complicated cases, in those where the powers of life have been previously injured, where bleeding cannot be used with boldness, and where stimulants are required, the exhibition of the tartar emetic in full doses is very hazardous. The mercurial treatment is to be preferred, from its greater safety, and, in this (form of) disease, more than equal efficacy." (P. 344.)

Dr. Stokes mentions change of position as a very necessary measure in prolonged cases of peripneumony, in order to relieve the diseased portion from hypostatic congestion. "In many cases where remedies seemed to have little or no effect, attention to position has been followed by a rapid recovery." When, after the cessation of fever and local irritation, the lung continues in a congested state, with copious bronchial secretion, the treatment recommended in the second stage of bronchitis (senega, squill, &c.) is often effectual.

The chief peculiarities in the treatment of typhoid pneumonia are stated to be these:

"1. That general bloodletting is to be used with extreme caution. 2. That the mercurial is in general to be substituted for the antimonial treatment. 3. That counter-irritation may be employed at an earlier period. 4. That the vital forces are to be carefully supported. 5. That as gastro-intestinal disease frequently complicates the pneumonia, close attention must be paid to the abdominal viscera. 6. That stimulants are to be used with greater boldness, and at an earlier period." (P. 350.)

The next section is misnamed "Perforating Abscess of the Lung," for it contains cases of purulent collections formed exterior to the lung, but afterwards perforating its tissue, and evacuated by the bronchial tubes. "Abscess perforating the lung" would be a less objectionable title. The following are the chief cases:—1. Abscess of the thoracic or abdominal integuments passing across the pleura by adhesion, and forming a fistulous communication with the lung. 2. Purulent collections in the serous membrane, opening directly into the lung. 3. Hepatic abscess perforating the diaphragm, and being discharged through the bronchial tubes.

Of these, the last is the most frequent. We lately saw a case in which a collection of pus between the diaphragm and the stomach communicated by fistulous openings with the latter organ, and through the diaphragm and pleura with the lung: pure pus had been voided the day before death, apparently by both coughing and vomiting.

Before we enter on the section of Dr. Stokes's book devoted to "Tubercle of the Lung," in which he treats chiefly of the diagnosis and treatment, and in a very masterly way, we would shortly notice some of M. Andral's notes on the nature of tubercle. He approves of the term "*accidental production*," as applied by Laennec to tubercle and similar results of alteration of the nutritive process of tissues, and, more strongly than in his former works, deprecates the attempts of the Broussaians to refer these lesions merely to *irritations of secretion*. Irritations of every degree may exist without developing tubercle; and tubercle is produced where there is no proof that irritation has preceded it. Inflammation may no doubt accelerate or determine the production of tubercle, where that modifying influence which is the real cause of tubercle is already present; but it is then only an accessory or secondary agent, and the condition which is essential is yet a subject for future research. It is well known that M. Andral was the first among modern pathologists who adopted the opinion formerly expressed in the Phthisiologia of Morton, that tubercle is a peculiar kind of morbid secretion from the blood, and that a depraved condition of this fluid, arising from various causes, is the chief element in the tuberculous diathesis. This view, so important in its tendency to direct attention to the constitutional origin as well as the local development of tubercle, has been subsequently adopted by Drs. Carswell and Clark; and the latter physician has prosecuted the subject a step further in the attempt to point out the functional disturbances which precede and accompany the development of tuberculous diseases.

With regard to the nature of tubercle itself, it seems to have been Laennec's decided opinion that it is endowed with a special vitality, that it grows by intussusception, and softens by its own intrinsic properties. This would imply that it is organized, yet Andral and others have failed to discover in it either vessels, laminæ, cells, or fibres, but merely an homogeneous substance, like those amorphous concretions which result from a kind of solid precipitation from the animal liquids. But M. Kuhn has recently announced that he has, through the microscope, discovered in the early condition of tubercle a distinct organization in an extremely fine filamentous web, of gelatinous appearance, which contains an agglomeration of yellowish particles, and contained in a muco-membranous envelop. These fine filamentous threads M. Kuhn calls the *tuberous tissue*, believing it to be the matrix of the albuminous particles which form within it, and constitute the bulk of tuberculous matter. It ramifies afresh through the particles thus produced, and forms more around them. In the early stage of tubercles, these particles float in a clear mucus; but afterwards this mucus is absorbed, the particles approximate, and form a crude tuberculous mass. In the early stages of phthisis, when the expectoration appears to the naked eye to be simply that of bronchitis, M. Kuhn asserts that he has, with the microscope, discovered in the sputa portions of the same filamentous tissue. Should this be confirmed, it would, as M. Andral observes, be a precious discovery for diagnosis; but these researches do not appear to have been repeated by any one. The notion of Broussais, that pulmonary tubercles are degenerated lymphatic glands, is quite obsolete. In regard to it, M. Andral remarks that he has sometimes found lymphatic vessels in the lung distended with pus and a matter like tuberculous, without giving anything of the appearance of pulmonary tubercles. In noticing the opinion of Dr. Carswell, that pulmonary tubercle has its seat in the air-cells, being secreted chiefly by the mucous membrane, at first dense and grey, afterwards more liquid and yellow, M. Andral observes that, as tubercles unquestionably are produced in the substance of other organs, it is most probable that they are so in the lung likewise. He considers the cases cited in support of Dr. Carswell's opinion as exceptions, the more general rule being that it is within the very texture of organs, in the cellulo-vascular tissue, which is the source of nutrition and secretion, that tubercle is chiefly formed. This is also the opinion of M. Lombard. For our own part, viewing yellow tuberculous matter, and the various grey indurations that precede it, as nothing else than a degraded albuminous lymph, possessing a very low vitality or totally destitute of it, and therefore incapable of further organization, we see no reason why it should not be produced in all situations where coagulable lymph may be produced. So, in truth, where, under the influence of inflammation, we find fibrinous lymph occasionally effused, on serous and mucous membranes, in cellular tissue, within the parenchyma of organs, nay, even in clots of blood, there also we have seen tuberculous matter make its appearance under those circumstances which cause its production.

On the connexion of inflammation with tubercle, M. Andral confesses that, the more he has observed and studied the subject, the nearer is he brought to the opinions of Laennec. Considering tubercle to be the result of a peculiar modification of nutrition and interstitial secretion, he sees no reason that irritation should be an essential element of the process, any more than it is necessary for common nutrition or for the secretion of bile. Irritation, in all its forms and degrees, often exercises a great influence on the production of tubercles, but only as an occasional cause: it cannot produce tubercles without a predisposition; but the predisposition may lie dormant until irritation of some kind brings it into play. This view still falls short of that of Laennec, who, referring the origin and growth of tubercles to a sort of seed-like germination of intrinsic vitality, could not suppose them to be under the influence of vascular action, as secretion and nutrition are. We question whether M. Andral's mind is settled on this point, and whether his disposition to concede more to the dead (Laennec) than to the living (Broussais) may not bias him. The individuals most disposed to this modification of nutrition are, according to M. Andral, those in whom the "organic development" has not been complete; those, in other words, who manifest signs of the lymphatic temperament. Tubercles may, however, be produced in cases where there are no such external signs of the predisposition; but, "*that the predisposition exists even in these cases, is proved by the appearance of tubercles, which would not take place without it.*" (*Notes*, p. 221.) Such a specimen of reasoning in a circle is not to be found in M. Andral's earlier works. We can more fully go with him in the reasonableness of the next remark: "Although irritation does not always precede tubercles, yet it constantly follows them. In every case where an organ has been invaded by these products, there takes place around them a reaction, the result of which is an inflammatory process, and the end of which is the expulsion of the tubercles." (*Ib.*)

Dr. Stokes far exceeds all his predecessors in the minuteness and accuracy of his observations on that touchstone of diagnosis, the signs of the early stages of phthisis. We strongly recommend them to the attention of all our readers; for, whether young or old in the practice of auscultation, they will be sure to derive instruction from a careful perusal of them. Their minuteness may discourage many; but we can assure our hasty brethren that, although they may now and then make off-hand hits in other cases of auscultation, they never can become accurate in distinguishing the early stages of tuberculous disease, without being very patient and minute in their investigations. Dr. Stokes enumerates the physical signs of pulmonary phthisis as follows:—1. Signs of irritation; *a*, of the mucous membrane; *b*, of the air-cells; *c*, of the serous membrane. 2. Signs of solidification. 3. Signs of ulceration. 4. Signs of atrophy. 5. Signs referrible to the circulating system.

Under the head of "Signs of *Irritation*" are included all the bronchial rhonchi, feebleness of respiration, and dulness of sound. This is an instance of that tendency to Broussaian generalization which we have before noticed as one of the few defects in this work. They should rather be called signs of *obstruction;* for they all imply an impeded passage of air into the lungs, and it is far from certain that many of them are essentially connected with irritation. The mucous, the muco-crepitating, and sibilous rhonchi, with feebleness of vesicular murmur, and a shade of dulness of the clavicle or spinous ridge of the scapula, are the most common signs; but it is from their partial localization and permanency only that they derive their diagnostic value in phthisis.

"Simple bronchitis is seldom circumscribed, while that of the consumptive is commonly so: the latter begins in the upper portion of the lung, remains obstinately fixed in the air-tubes, gradually spreads downwards, and, while in its first stages in the lower lobe, is combined with tuberculous ulceration in the upper; it may be intense in the upper lobe, while the lower is altogether free, or engage the whole of one lung, while the other is scarcely affected. These are not the characters of ordinary bronchitis." (P. 392.)

But there may be a *general* deposition of tubercles, and consequent absence of localization of signs, as often happens in acute phthisis, which runs a rapid course. Partial feebleness of respiration, ascertained by comparison of the corresponding portions of the lungs, and of the upper and lower lobes, is an important sign, and should be tested by forced as well as ordinary respiration. Dr. Stokes says that there is, however, in many individuals, particularly nervous persons, a natural difference between the intensity of the murmur in either lung; that in the left being almost always the loudest.* The morbid sign would be distinguished from this by its being even more partial, and accompanied by dulness or some rhonchus. "In ordinary cases, the feebleness of respiration is almost always modified, and often removed, by a timely anti-

* This would seem the reverse of Dr. Gerhard's opinion, noticed in vol. iii. p. 188, of this Journal.—ED.

phlogistic and revulsive treatment; and there can be no doubt that in this way many a patient can be saved from impending consumption." (P. 396.) Dr. Stokes does not notice another sign of obstruction which Andral considers often available in the early stage of phthisis,—the increased sound of *expiration.* This sound is scarcely audible in the natural vesicular murmur, which is almost wholly composed of the sound of inspiration; but, when aggregated tubercles, or other causes, obliterate several of the bronchial tubes, the exit of air from the tissue becomes audible in various degrees; in some cases heard only during forcible breathing, but in others it is loud and whiffing, and almost masks that of inspiration. We think that the circumstance of dilatation of the air-cells, and perhaps also occasionally of simple bronchitis producing a similar phenomenon, impairs the value of this sign, unless there be also dulness on percussion. On this last subject Dr. Stokes gives no less than twenty-six different modes of dulness on percussion from tubercles, that he has repeatedly verified. Of these, the following are interesting specimens:—6th. Right clavicle and left scapular ridge dull. 7th. The converse of the last. 20th. Comparative dulness of one lung, with puerile respiration under the clavicle. 26th. General but incomplete dulness of both lungs, supervening on bronchitis, or, with crepitating râle, persisting to the fatal termination. In a few instances, dulness under the clavicles coincides with a bronchial respiration; and this latter sign is sometimes only audible in the erect posture. This is because the chest is then more raised, and the respiration more complete in the upper parts of the lung. The phenomenon of bronchophony, or vocal resonance under the clavicles, is wholly unnoticed as a sign of tubercles; yet we are sure that, in many cases, when distinctly louder on one side, and at the humoral end of the clavicle, it is entitled to some confidence. The vocal signs are altogether too much depreciated by Dr. Stokes.

In the use of percussion in these cases, particularly on the clavicles and scapular ridges, the most delicate mediate percussion with a single finger is to be preferred. In doubtful cases, Dr. Stokes recommends it to be practised also whilst the patient holds in a full inspiration. Dr. Williams had likewise, in the paper before quoted, advised that, in such cases, percussion should be performed during different degrees of the respiratory act. Sometimes a difference is perceptible only after a full expiration. The last-named writer states, moreover, that mediate percussion with the fingers of the flat hand, applied over a large surface, will often detect a dulness from scattered tubercles, when percussion on a single finger, which is exclusively employed by Dr. Stokes, fails to yield any indication; and our own experience corroborates the opinion of Dr. Williams. Dr. Stokes does not allude to the complication of tubercle with emphysema, which we have often found to render the results of percussion equivocal. The dilatation of the air-cells accompanying numerous scattered tubercles is generally superficial; and we have seen a case in which it caused the whole chest to yield a clear sound, although a vast number of tubercles of various sizes, and not a few small vomicæ, were found after death, within the lung, beneath this emphysematous surface. There is, however, usually an irregularity in the percussionary sound in these cases; and, by using strong as well as gentle percussion, a difference may be generally detected. It is this emphysema, more than the atrophy of the lung, as supposed by Dr. Stokes, that often renders the sound clear even in the last stages of phthisis.

Dr. Stokes offers little that is new on the subject of the Signs of Ulceration of the Lung. He trusts only to cavernous respiration and gurgling as signs of a cavity. He sets scarcely any value on pectoriloquy, and thinks that, if the ear be well accustomed to the other signs, this may be neglected. We think that he under-rates this sign as much as Laennec over-valued it. When heard (*with the stopper in the stethoscope,*) in any part of the chest where the respiration is naturally purely vesicular, and especially if it be of that perfect kind in which the voice seems to be spoken *into the tube,* whether it be accompanied with cavernous respiration or not, we consider it a sign of a cavity communicating with the bronchi, as certain as the signs trusted to by Dr. Stokes. It is true that it may be absent when they are present, especially when the cavities are not empty, or do not open freely into the bronchi; but we have known instances in which pectoriloquy was heard without any cavernous respiration. We have before hinted at the reason why Dr. Stokes, M. Andral, and others, do not sufficiently distinguish the value of pectoriloquy; they do not test it by the stethoscope with the stopper in, without which it is often impossible to distinguish it from morbid or even natural bronchophony. The latter phenomenon, which is extended over a wider space, is so much reduced by using the stopped stethoscope, that it can then be readily distinguished from true pectoriloquy, which, being produced in a circumscribed cavity, is still transmitted through the simply perforated cylinder.

Dr. Stokes makes some valuable remarks on the partial contractions of the chest which take place in phthisis, and which he believes to be produced in many cases by a wasting of the lung, and independently of the formation of cavities. M. Andral has expressed the same opinion. For measuring the upper parts of the chest with a view to detect these contractions, Dr. Stokes makes use of graduated spring callipers, one knob of which is fixed on the scapula, and the other below the clavicle. We have been in the habit of judging of the comparative size of the upper lobes of the lungs by inspection, not only from in front and behind, but also from *above,* by looking down at the patient's chest from behind the top of his head; and we have often thus discovered a defect in symmetry that was not perceptible by ordinary inspection. But Dr. Stokes's callipers will probably give us more accurate results.

We regret that our limits do not permit us to quote the very interesting observations which Dr. Stokes gives on the Varieties of Phthisis; the perusal of which, however, we earnestly recommend to the attentive perusal of all practitioners. This we do the more strongly, because we are convinced, from much observation, that diseases, considerably differing from each other in pathological character and origin, and, in relation to treatment, are, both by writers and practitioners, still confounded together under the name of pulmonary tubercle and phthisis.

In discussing the treatment of phthisis, Dr. Stokes considers it under the two heads, *curative* and *palliative ;* and, although he admits that the latter is that which we must generally follow, yet he doubts not "that, as medicine advances, the cures of consumption will be much more frequent, its nature will be better understood, its first stages more commonly recognized, and the disease prevented from proceeding to incurable dis-organization." Our hopes, if not our confident expectations, are the same. With relation to the treatment, Dr. Stokes divides the cases of phthisis into two classes, the constitutional and the accidental. This is very much the division of Professor Alison and Dr. Williams, and that formerly held by M. Andral, who now, however, seems to consider, with Drs. Clark, Carswell, and others, that the constitution is involved in all cases. In constitutional cases, "tubercle supervenes either with or without precursory irritation in persons strongly predisposed to it by hereditary (we would add, or acquired,) disposition or original conformation. In these the disease is generally rapid, invades both lungs, and is complicated with lesions of other systems. The disease is constitutional, and the affection of the lung, though the first perceived, seems but a link in the chain of morbid actions. In the second, we meet the disease in persons not of the strumous diathesis, and who have no hereditary disposition to tubercle. The disease results from a distinct local pulmonary irritation, advances slowly, and *the digestive and other systems shew a great immunity from disease.* In both cases, we may effect a cure; but this result will be more often obtained in the latter than in the former class." (P. 438.)

Four forms of incipient curable phthisis are noticed by Dr. Stokes,—the *Localized Bronchitic*, the *Tracheal*, the *Hæmoptysical*, and the *Pneumonic* varieties. Dr. Stokes thinks that many cases of the first can be cured, if no time be lost. Besides confinement and rest, with mild farinaceous diet, a sedative for the cough, and one bleeding at first, if the pulse be inflammatory, leeches in small numbers are to be repeatedly applied, alternately below the clavicles and in the axilla of the affected side; and, when these have diminished the dulness and the stethoscopic signs, a blister of the size of a dollar is to be applied, about every three days for several weeks, under the clavicle and over the scapular ridge: the blister may then be converted into a superficial issue, by dressing it with a disc of felt anointed with mercurial and savine ointments; the regimen may then be improved. Friction with the turpentine liniment, and inhalation of the vapour of warm water impregnated with twelve or fifteen grains of extract. cicutæ may now be employed; and, in mild weather, exercise on horseback. A milder climate and frequent changes of situation complete the recovery. The hæmoptysical variety is to be treated on the same principles, but bloodletting, general and local, must be used more freely to restrain the hæmorrhage. The tracheal and pneumonic varieties will generally require the use of mercury in addition to other measures.

"Mercurial treatment of incipient phthisis" is a title which will startle many of our readers; nevertheless Dr. Stokes assures us that not only he, but Drs. Graves and Marsh, "have treated with mercury several cases of incipient pulmonary disease, which would in all probability have ended in phthisis." He admits that the cases are too few to establish the treatment, and that "the remedy is a two-edged sword, and its exhibition must not be lightly attempted." He gives two cases of permanent and one of temporary recovery, the latter dying some months after, tuberculous. The signs of these cases were, (besides the general symptoms,) dulness on percussion, and feebleness of respiration, with obscure rhonchus under one clavicle, all of which were removed when ptyalism was produced. We agree with Dr. Stokes in supposing that, where tubercles exist, they often produce physical signs only by the irritation and subsequent change which they may cause in the lung, the latter may be removed by treatment, and the physical signs with them; but, we are not sure that the tubercles go also, and therefore that the subject is safe. Years of the subsequent history are required to establish this. Still, whether the treatment cure or only intercept and arrest this formidable disease, the profession is deeply indebted to the talents and industry of Dr. Stokes, for his rational efforts to improve the diagnosis and treatment of incipient phthisis.

Treatment after excavation has formed, has in some cases prolonged life for many years, and in a few there has been a complete recovery. The principal remedy was the seton, with frequent changes of air, or sea-voyaging, resorting in winter to a temperate and equable climate; and, in this respect, "the Cove of Cork is surpassed by few places." The less medicine the patient needs to take the better. "So long as a drain from the chest does not weaken, it is clearly useful, and all the other means should be calculated to give enjoyment to the mind and to strengthen the body."

The palliative treatment, although that commonly called into requisition need not detain us, as the observations of Dr. Stokes are those of most judicious practitioners. He enjoins great caution in adopting measures to check expectoration, which is the natural relief of the lung. The inhalations of iodine, chlorine, and tar, act in this way and are consequently hazardous. "They have no specific action on tubercle, but, by arresting purulent secretion, they cause a more rapid development of the disease." Our own experience accords much with this. It is not so with the internal use of iodine; but of this we have no room to speak, and we find that we must postpone Dr. Stokes's interesting Section on Diseases of the Pleura, and the remainder of M. Andral's "Notes," with the whole subject of the Larynx and Trachea, to a future Number.

The unusual length of this still unfinished article sufficiently indicates our opinion of the importance of the subjects, and the ability of the authors commemorated in it. The notes and additions of M. Andral are the contributions of an active and philosophic spirit, seeking nature beyond his predecessor's track, but still giving homage to the master-mind before him. Those of M. Meriadec Laennec are of a much inferior order; their chief goodness consisting in the recapitulations of the text, and the extracts of the opinions of other writers, chiefly French; for, except in some imperfect notices of the additions of the English translator, there is nothing taken from this side of the channel. But the book of Dr. Stokes is altogether of a higher order, and is not only a most important and valuable contribution to our knowledge in the diagnosis and treatment of thoracic diseases, but it is a work which we would cite as a proof of the generally improved state of medicine, and as a production justifying the belief which we expressed at the beginning of this article, that medicine is really assuming the character of an inductive science. We recommend it, in the strongest terms, to all classes of our readers. We have only one admonition to give the author,—viz. to pay more attention to the literary execution of his next volume.

